# Security risk models against attacks in smart grid using big data and artificial intelligence

**DOI:** 10.7717/peerj-cs.1840

**Published:** 2024-04-26

**Authors:** Yazeed Yasin Ghadi, Tehseen Mazhar, Khursheed Aurangzeb, Inayatul Haq, Tariq Shahzad, Asif Ali Laghari, Muhammad Shahid Anwar

**Affiliations:** 1 Computer Science and Software Engineering Department, Al Ain University, Abu Dhabi, United Arab Emirates; 2 Department of Computer Science, Virtual University of Pakistan, Lahore, Pakistan; 3 Department of Computer Engineering, College of Computer and Information Sciences, King Saud University, Riyadh, Saudi Arabia; 4 School of Electrical and Information Engineering, Zhengzhou University, Zhengzhou, China; 5 Department of Computer Science, COMSATS University Islamabad, Sahiwal Campus, Sahiwal, Pakistan; 6 Software College, Shenyang Normal University, Shenyang, China; 7 Department of AI and Software, Gachon University, Seongnam-si, Republic of South Korea

**Keywords:** Smart grid, Big data, Cybersecurity, Artificial intelligence, Machine learning, Deep learning, Cybersecurity risks, Automated distribution network, Blockchain, Methods

## Abstract

The need to update the electrical infrastructure led directly to the idea of smart grids (SG). Modern security technologies are almost perfect for detecting and preventing numerous attacks on the smart grid. They are unable to meet the challenging cyber security standards, nevertheless. We need many methods and techniques to effectively defend against cyber threats. Therefore, a more flexible approach is required to assess data sets and identify hidden risks. This is possible for vast amounts of data due to recent developments in artificial intelligence, machine learning, and deep learning. Due to adaptable base behavior models, machine learning can recognize new and unexpected attacks. Security will be significantly improved by combining new and previously released data sets with machine learning and predictive analytics. Artificial Intelligence (AI) and big data are used to learn more about the current situation and potential solutions for cybersecurity issues with smart grids. This article focuses on different types of attacks on the smart grid. Furthermore, it also focuses on the different challenges of AI in the smart grid. It also focuses on using big data in smart grids and other applications like healthcare. Finally, a solution to smart grid security issues using artificial intelligence and big data methods is discussed. In the end, some possible future directions are also discussed in this article. Researchers and graduate students are the audience of our article.

## Introduction

Around the world, the idea of a “smart and sustainable city” is used mainly for two reasons. To achieve this goal, “smart” power networks prioritizing renewable energy sources are being built, as they are crucial to managing energy better. The second goal is to encourage less driving habits, which will lower carbon dioxide emissions. Information and communication technologies (ICT) offer several benefits, but they are not a goal in and of them. A wise and sustainable urban policy is necessary to lessen the city’s adverse effects on the environment and enhance the quality of life for its citizens (Paul et al., 2014). Cities that use ICT are more innovative, sustainable, and desirable places to live. This is a challenging subject because cities are expanding and becoming crowded as more people move there.

**Figure 1:**
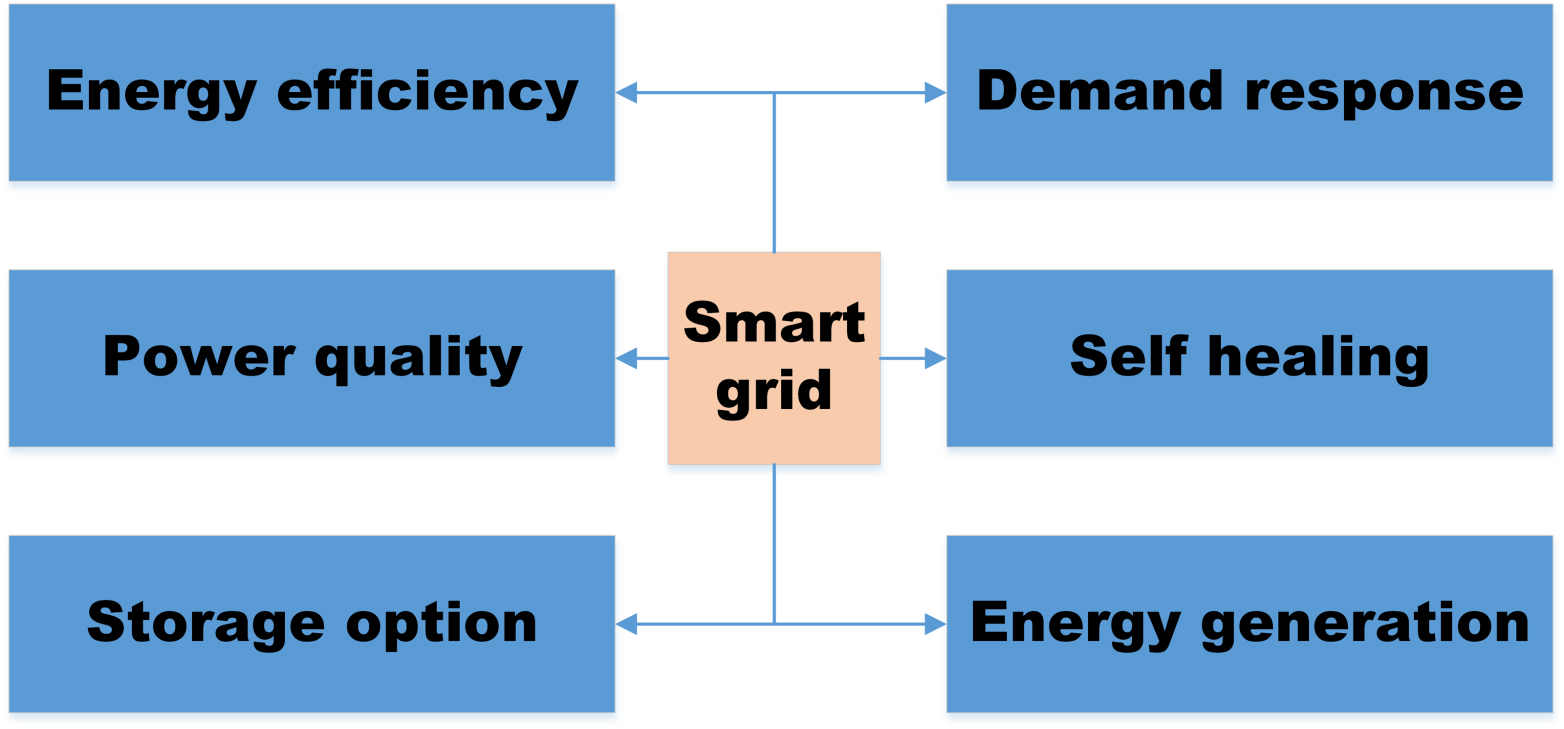
Security threats during implementation of smart grid (Judge et al., 2022).

Electric companies are switching to a system that is more advanced and automated. Suppose energy companies want to provide a continuous electricity supply in the face of rising demand for digitalized, linked, and integrated activity across all disciplines. In that case, they must prioritize efficiency and renewable resources (Mistry et al., 2020). Cybersecurity dangers and attacks are rising due to the electrical grid’s increased interconnectivity and “smartness.” This is accurate quite apart from the advantages of having more connections. Innovative grid technology will make the benefits of distributed power and renewable energy more accessible and more direct for networks and customers (Aitzhan & Svetinovic, 2016). It will be simple to control the two-way flow of electrical energy using a smart system. It will also make monitoring, managing, and supporting resources easier at the distribution level. Power companies can utilize their existing infrastructure more effectively and lessen the need to build new power plants using intelligent grids. Smart grids are self-sufficient and improve the effectiveness and efficiency of managing electrical power (Zheng et al., 2018). The system must be flexible for the coexistence of distributed and centralized renewable energy sources (Hassija et al., 2020). Reducing the number of vulnerable targets, such as large power plants, will result in a considerable change in system security in terms of supply and the case of a disaster. System modernization will dramatically minimize greenhouse gas emissions from existing power plants by removing issues with erratic supply and minimizing the need to invest in a central fossil-fuel generator. This is because it will encourage the more significant distributed generation and the development of dependable locations for renewable energy sources, such as solar and water. Figure 1 explains the security threats during implementation.

One way to assess a user’s vulnerability is to examine security concerns in embedded systems and smart grid (SG) technology. For instance, by looking at cybersecurity from the game theory perspective, we can decide on monitoring and protection choices based on how interactions are affected by formalized market incentives. However, security can be increased by taking charge from the viewpoint of planned cyberattacks. In conclusion, organizations in the energy sector keep an eye on cyber security while ensuring the proper operation of crucial power supply components. This guarantees the dependability of the modernized grid. The development of new smart optimization techniques, including genetic algorithms, neural networks, game theory methods, reinforcement learning, and vector support machines, has primarily been responsible for improved electrical network reliability, safety, and effectiveness. Scientists have been able to look into how security systems react to shifts in the energy market using methods from the earlier study. As a result, modern SG control and monitoring systems make it easier to find crucial infrastructure parts quickly.

**Figure 2:**
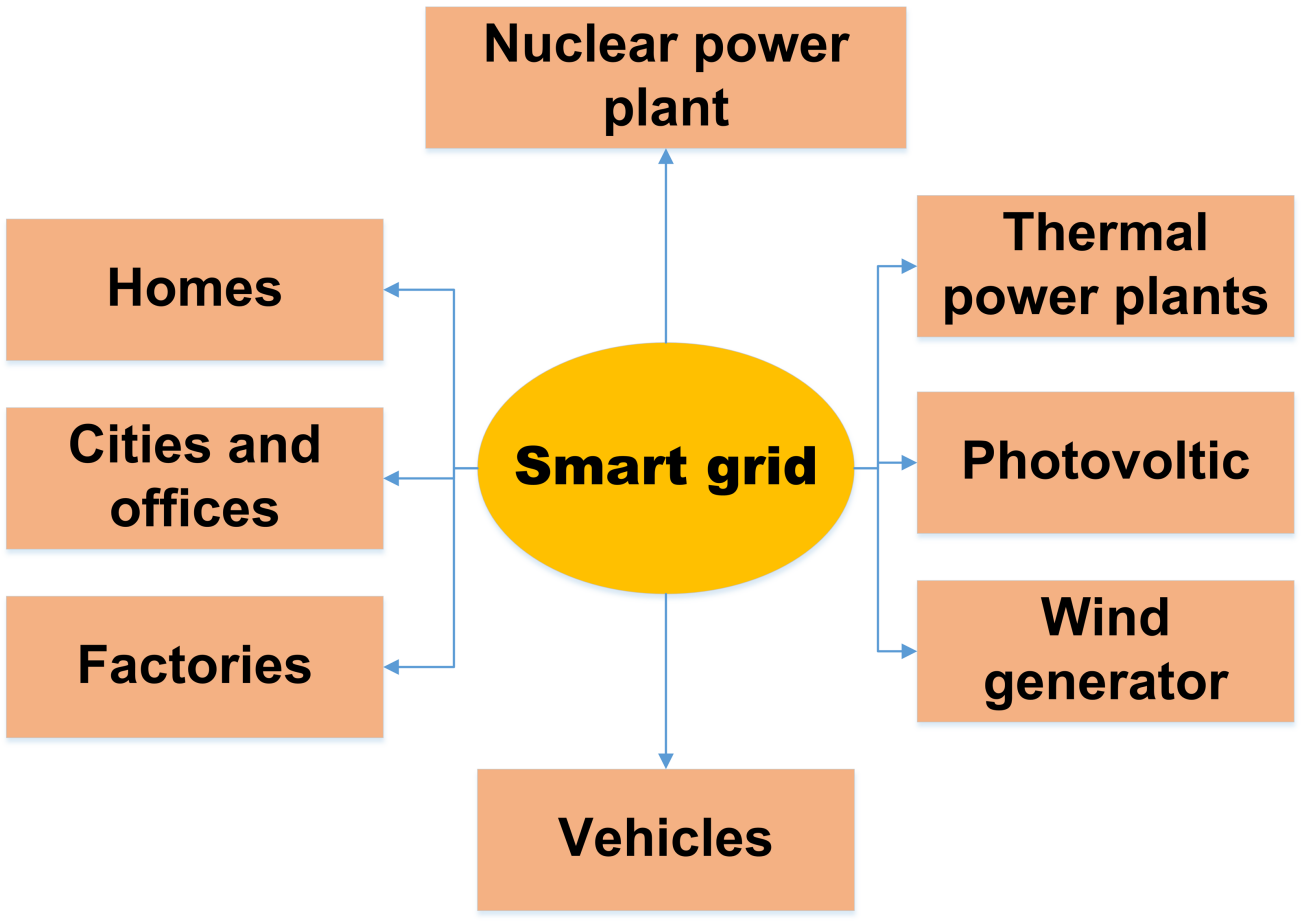
Relation between IoT in smart grid (Alavikia & Shabro, 2022).

One of the most creative advancements in communications is the Internet of Things (IoT). The term “Internet of Things” is widely used to describe a network of connected technological devices that share data. The expected difficulties in switching from traditional energy networks to new smart grid systems could be addressed *via* the Internet of Things (Parimi & Chakraborty, 2020). This is because IoT offers distributed computing and bidirectional networking. A large number of distributed renewable energy sources, live, real-time data communication about tariff increases and energy use between consumers and service providers, infrastructure to collect and transfer statistics about grid parameters for analysis, and the capability to act in response to these analyses are all necessary components of a smart grid (Parimi & Chakraborty, 2020). The smart energy grid generates much data and information that must be transmitted, processed, and stored for efficient decision-making (Yeboah-Ofori, Islam & Brimicombe, 2019). Because it has so many uses, the Internet of Things might be a perfect fit for the smart energy system. The Internet of Things may make moving away from the traditional power grid and toward the more advanced smart energy system more accessible (Tuballa & Abundo, 2016). This is because IoT has higher precision and competency. After all, it is proactive and intelligent. We have optimism for a solution because of the potential of IoT to improve power quality and dependability, two of the biggest problems with the power system. By including intelligent information-processing capabilities during the flow of electricity from the service provider to the customers, energy monitoring infrastructure and innovative metering technologies can help transform a traditional power grid system into an intelligent grid system (Yeboah-Ofori, Islam & Brimicombe, 2019). This integrated system may gather information on energy use, voltage levels, current flows, and phase angles, among other things. Improved energy grid control is made possible by trying to cut the Internet of Things technology’s capacity to gather and intelligently analyze enormous amounts of data. Figure 2 explains the relationship between IoT and the smart grid, and Table 1 illustrates a list of abbreviations used in this article.

**Table 1:** List of abbreviations.

Abbreviations	Full form
IoT	Internet of Things
AI	Artificial intelligence
SG	Smart grid
SB	Smart buildings
LTE	Long-term evolution
ML	Machine learning
SGMS	Smart grid management system
HTTPS	Hyper Text Transfer Protocol
WAN	Wide-area network
SCADA	supervisory control and data acquisition

Current load forecasting (LF) approaches were analyzed in Alavikia & Shabro (2022) to determine the most effective course of action for particular situations or scenarios. Time, inputs, outputs, scale, sample size of data, type of error, and value were compared between these approaches. Long-term load forecasting (LTLF) was dominated by regression and multiple regression, whereas STLF and VSTLF were dominated by machine learning (ML) approaches such as artificial neural networks (ANN), support vector machines (SVM), and time series analysis with ARIMA and ARMA.

A hybrid computer method for STLF that considers stochastic load demand was proposed by Alavikia & Shabro (2022). With this method, three models are combined into one: LGBM, XGB, and MLP. In the stacked XGB-LGBM-MLP strategy, the MLP network generates final forecasts using meta-data from XGB and LGBM models. Several case studies were used to evaluate this approach.

The author Nasir et al. (2021) employed the multi-space collaboration (MSC) framework to optimize the selection of models. The possibility that the MSC will choose the best model was boosted by using a space separation technique for model selection on subspaces. Their approach removed low-potential subspaces between iterations to concentrate on superior parameter domains. As simulations and real-world case studies demonstrated, the MSC framework showed excellent robustness and outperformed previous meta-heuristic algorithms.

It is challenging to choose the best ML and deep learning (DL) algorithm for electricity demand forecasting among the many available LF techniques, given the significance of LF techniques in preserving the dependability, stability, and efficiency of smart grids (SGs), particularly in predicting energy demands. Modern LF techniques and their uses in SGs were thoroughly evaluated to overcome this challenge.

The management of power generation infrastructure, the use of data acquisition and control systems to manage transmission and distribution operations, the implementation of advanced metering infrastructure, and the monitoring of carbon footprints and the environment are just a few ways that IoT technologies can have a significant impact on smart energy grid systems. The cyber risks associated with the traditional centralized SCADA system can be reduced by utilizing current cloud and edge computing technologies (Alavikia & Shabro, 2022; Nasir et al., 2021). This allows remote energy resources to be managed and monitored without centralized control (Mistry et al., 2020). Other intelligent things like utilities, homes, buildings, and cities may connect with the IoT-enabled smart grid. This makes it easier to run and control the electrical grid. To achieve this, you must have computer literacy and a plan for using your resources. Although the Internet of Things improves the efficiency of monitoring and managing energy systems, an IoT-based smart grid is challenging to install. Cyber attackers in the Internet of Things might target operational, financial, and system security. Numerous examples of these kinds of losses can be found in Mistry et al. (2020):

• Localized and large-scale power outages.
• This suggests that energy providers and the electricity industry would face substantial financial losses.
• Online sharing of personal data puts users at risk for identity theft and other social harm.
• The functionality of Tran’s reactive energy systems was lost.
• IoT technology can enhance several components of the energy infrastructure, including power generation, transmission networks linked to SCADA, distribution networks, monitoring of emission particles, and smart homes and buildings. Modern IoT technology called fog computing can be used to improve and manage a transmission network based on SCADA. This opens up a lot of possibilities. Most smart home devices are fully automated due to the Internet of Things’ role of big data in different fields like healthcare.

### Motivation for this study

According to this study, secure models are necessary for the smart grid’s security. It is the basis for any security architecture and technology developed in a smart grid and improves the system’s dependability and resistance to cyberattacks. The electric grid’s adaptability, reliability, dependability, and productivity can be improved by using big data to make decisions about its management and operation. Smart grids are being built to handle more complex electricity generation and distribution. They are powered by cloud-connected technologies that use artificial intelligence (AI).

### Contribution of the study

Attacks are common, and it is important to consider who the attacker is, what vulnerabilities are used, what vulnerabilities are targeted, and the consequences of the attack. Security flaws occur when the confidentiality, integrity or availability of information, systems or other services are at risk. Each cybersecurity incident poses a different threat to an individual or organization’s systems and networks.

In this study we see architecture and components of smart grid and also study smart grid security infrastructure in big data and artificial intelligence. We also study different types of applications in which big data and AI playing very imoportant role and most common are healthcare. AI and big data teacniques are playing very important role to protect any sytem and most of the attcks on system can be reduced due to these teachniques. We also study different types of attacks on smart grid like scanning, exploitation black hole,and cybersecurity attcaks.We also study different types of machine learning,deep learning and AI teachniques and also some security models that are used against these attcaks.At the end we study different types of challenges that are faced to implements AI and big data teachniques in smart grid.

### Organization of the study

**Figure 3:**
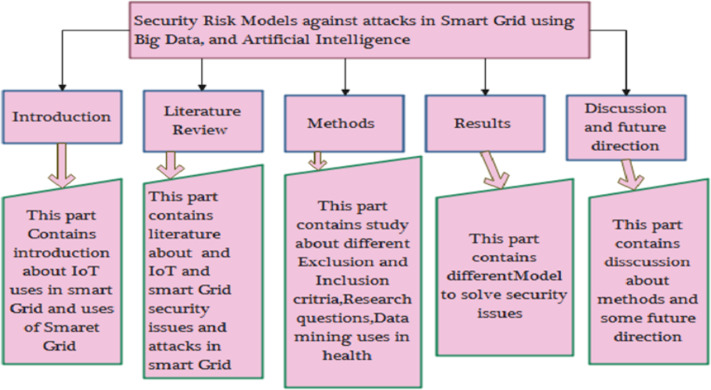
Organization of this study.

The remaining article is organized as follows. Section ‘Related work’ describes the related work in detail. Section ‘Methods and Materials’ discusses the methodology in which research questions, exclusion and inclusion of AI, and big data techniques and their use in health are concerned. Section ‘Results’ discusses the results of the research questions, and Section ‘Risk Modeling Techniques’ concludes the work. Figure 3 shows the organization of the article.

## Related work

The size and shape of the smart and sustainable city need to be determined through thorough research before it can be built. It is a complex system; therefore, all essential parties must be involved, including cities, businesses, and citizens. A “smart and sustainable city” seems good (Nasir et al., 2021). Using information and communication technology to improve and optimize city operations benefits human behavior, society, and the environment. Cities are using digital apps all around the world to make them smarter. Digitalizing and wising a city is not a goal in and of itself, though (Lopez, Rubio & Alcaraz, 2021). Technology is just one tool available in the digital age for enhancing cities’ affordability, mobility, and public involvement. People must alter their collaborative practices with a focus on public engagement to make the city more environmentally friendly and pleasant (Kakran & Chanana, 2018).

In 2025, 4.6 billion people, or 58% of the world’s population, will live in cities. In developed countries, this percentage will increase to 80%. By 2050, 75% of the world’s population will live in cities, considerably increasing their population density. A few difficulties with urban are overcrowding, pollution, climate change, lack of access to energy, and related problems. Lighting, heating, and transportation account for more than 65% of all primary energy used in urban areas, and these three industries also account for around 70% of all greenhouse gas emissions. The future city will need specific planning to deal with challenges like climate change and declining air quality (Asif et al., 2022). Many modern cities prefer energy management systems over other options. Due to the additional money that cities and their residents must spend due to climate change, the energy crisis is a severe problem. “Smart grid” and “smart city” are frequently used interchangeably when referring to electricity. It is possible to track the energy usage of every building in a neighborhood or city using smart meters and other sensors, from homes to factories. The authors in Kakran & Chanana (2018) collected data that helps keep systems operating smoothly by turning off inactive devices during peak hours and gives users helpful insight into their behaviors.

It is still possible to control power production and usage as efficiently as possible using this consumption data, decentralized electricity production from renewable sources, and electricity storage. Electricity can be generated during the day and delivered to homes at night while companies are closed by putting photovoltaic panels on top of commercial buildings. Electric vehicles have two power options: they can produce energy during high demand or store it later (Hasan et al., 2022a). The dependability of smart grids depends on communication application control systems’ trustworthiness, safety, and usability (Mufana & Ibrahim, 2022). Big data is the practice of using a lot of data to find helpful information that can help guide corporate decisions through electronic systems and networks. The big data architecture and framework show how data, networking, software, and hardware work together to accomplish this one goal. All devices send data using an internet protocol as a result of the integration of ICT, which raises security concerns. However, this protocol has security holes that the wrong parties might use against it (Goudarzi et al., 2022). The most important part of the security system for the smart grid is the CIA. Table 2 illustrates some AI techniques with advantages and disadvantages.

**Table 2:** AI methods used in smart grid.

AI Technique	Advantages	Disadvantages
ANN	When compared to other AI systems, ANN offer more clarity. AI is a discipline within the field of technology that uses a multi-step method to examine data in order to find possibly unexpected patterns while also integrating different educational philosophies.	Moreover, it needs more processing power and is at risk for flooding. The process of developing the model includes research based in empirical data.
SVM	The model is prevented from achieving a high degree of accuracy by modifying control parameters in ANN. This approach works best when the dataset has distinct and well-defined groups. By employing the kernel technique, learning a subject can be completed quickly and simply.	This strategy is not the best for managing large datasets due to its complexity. It is not possible to apply this strategy in situations where there is overlap across groups. Furthermore, the testing step requires a significant amount of time to complete.
ANFIS	An AI-generated neuro-fuzzy system combines the learning powers of ANN with fuzzy systems to create logic based on fuzzy rules and adjust its parameters. As a result, the system can operate more efficiently. As so, this addresses the underlying issues that have delayed the progress of fuzzy system development.	The number of calculations that must be done increases with the initial number of fuzzy rules that are applied, especially when more fuzzy rules are added.

**Figure 4:**
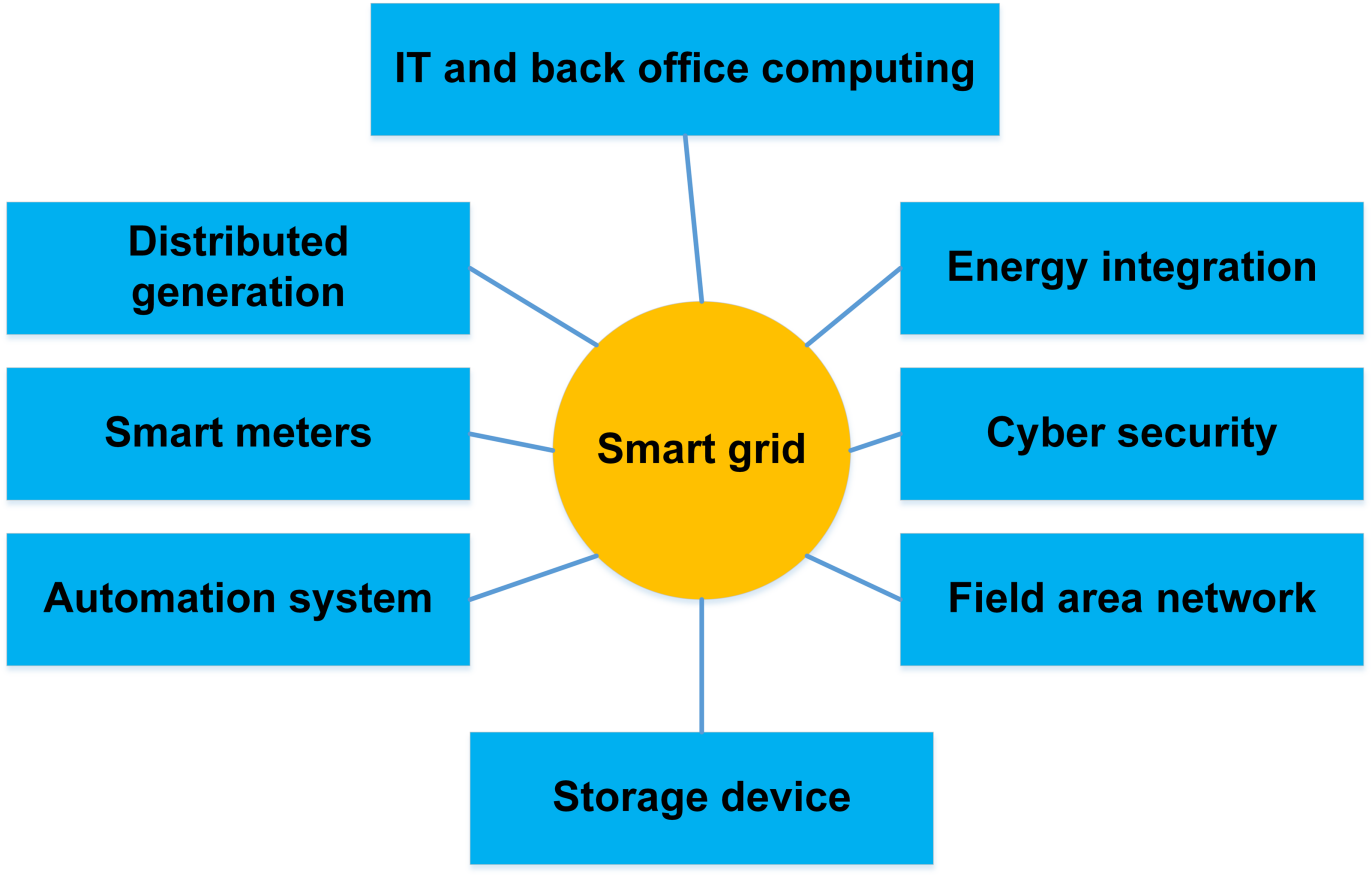
Smart grid systems and architecture (Judge et al., 2022).

The development of the SG, a highly secure, reliable, and environmentally friendly national power grid system, is driven by concerns about greenhouse gas emissions like carbon dioxide (CO2) and the need for more reliable and efficient power transmission and distribution (Yeboah-Ofori, Islam & Brimicombe, 2019). In conclusion, an SG transmits data in both directions to transfer electricity from generators to end users. It monitors and controls rising devices in homes and workplaces to make them more reliable, transparent, and energy-efficient (Ali et al., 2020). Modernizing the aging infrastructure of the power system is the goal of developing a smart grid. It automatically keeps track of the essential parts of the system, protects against damage, and enhances its functionality. Existing SG technologies are used in intelligent domains and connected situations, including energy distribution, communication networks, energy metering, and energy trading. Making the grid more dependable, secure, and private is the main aim of the traditional approach to supplying people with electricity. Until recently, many thought better communication and monitoring technologies made the electrical sector more dependable (Zheng et al., 2022). However, grid cybersecurity becomes more crucial as the grid becomes more interconnected. Electrical grid security aims to protect, prepare for, recover from, respond to, and lessen the effects of unforeseen natural disasters or cyber-system calamities. The most important solutions for guaranteeing the total security functionality of SG technology get more complicated as more security systems, protocols, and algorithms are integrated into it. Figure 4 explains the smart grid architecture, and Table 3 illustrates some key challenges of big data in the smart grid.

**Table 3:** Summary of key challenges to apply big data to smart grid.

References	Challenges	Possible impact	Potential solution
Succetti et al. (2023)	Data volume	It is necessary to increase both the capacity of the machine and the storage space it offers.	Reduce in complexity, parallel computing, processing at the edge, cloud computing,
Mazhar et al. (2023a)	Data quality	Incomplete information, incorrect decision	The process of preparing data for analysis using nonlinear and conditional models
Yadav et al. (2022)	Data security	Susceptible to harmful attacks, compromising the security and privacy of clients, and having the power to affect business decisions and financial transactions.	Data anonymization
Hasan et al. (2022b)	Time synchronization	Performing operations, interpreting data, and conducting historical analysis choices that are in direct opposition to the course of history	With the help of radio clocks or satellite receivers, it is possible to coordinate the operation of multiple devices concurrently.
Mitra et al. (2023)	Data indexing	Due to the complexity of the algorithm and the long period required for processing	Introduce innovative approaches to indexing, such as R-trees, B-trees, and Quad-trees.
Judge et al. (2022)	Value proposition	The lack of acceptance from stakeholders is causing the adoption of big data to be slower than expected.	The process of giving an amount to the technical and economic advantages that will be gained by the consumer, the system operator, and the utility supplier as a result of the implementation of the solution.
Fawzy, Moussa & Badr (2022)	Standards and regulation	In addition to a delay in deployment, there were issues with the interfaces that connect the various computers, storage, and processing systems.	Standards should guarantee the supervisory features of data sharing and exchange, and regulatory organizations should describe these aspects.

The term “smart grid” is a concept in which the power grid’s generation, transmission, and distribution are all combined into a single entity. In other words, the system becomes more intelligent, effective, and secure with the addition of a smart grid (Ghiasi et al., 2023). The importance of renewable energy sources is rising on a global scale. Because of this, “clean energy” and “smart energy” are synonyms (Kakran & Chanana, 2018). The term “smart grid” was first used in 2003. Michael T. Burr used it for the first time in an article. He discussed how system flaws could be found and fixed to enhance the movement of energy from its source to its destination (Ghadi et al., 2023). The design objectives of the SG that are now realizable as a result of this SG idea are shown in Fig. 4. It succeeded because a new feature that simplifies processes was being used effectively. The smart grid is built using the national security system and centralized control. To do this, distributed computer agents are used to construct an identity power system network, transmission device monitoring and diagnostic, grid computing, manage the complete power system as a hybrid adaptive power system, and manage the power system as a whole (Zheng et al., 2022). A review of how a smart-grid utility implemented the NIST Cybersecurity Framework is given within the framework of a case study. The study’s main goal is to examine how closely cybersecurity practices follow National Institute of Standards and Technology (NIST) standards, focusing on topics including risk assessment, incident response, and continuous monitoring (Shackleford et al., 2015). Through this analysis, the case study makes an effort to evaluate these measures’ effect on grid resilience, operational efficiency, and the overall defence against cyber threats. Because of their high levels of interconnectivity and reliance on digital technology, smart grids face substantial cybersecurity challenges that require careful thought and consideration. The multi-criteria decision-making (MCDM) approach provides a systematic framework for evaluating and contrasting different cybersecurity choices according to several different factors. These requirements include influence on grid performance, cost, practicality, regulatory compliance, comparability with current systems, and efficacy. Decision-makers can make well-informed choices by applying MCDM techniques to evaluate trade-offs between various cybersecurity options statistically. Applying the MCDM-AHP technique enables decision-makers to make informed decisions about cybersecurity options in smart grids. This methodology addresses the complexities and uncertainties related to cybersecurity by facilitating the thorough assessment of numerous issues. Ultimately, it helps choose the best and most appropriate defenses against cyberattacks on the smart grid system. Computers and mobile devices may be used in smart buildings to monitor temperature more efficiently, control security, and perform maintenance. SG uses IoT to coordinate building activities. Building management systems, IoT sensors, AI, and machine learning are all used in intelligent buildings. A few such potential technologies include AI and ML (Mazhar et al., 2023a).

Building automation and management systems, or SGMS for short, are required to accurately keep track of the amount of energy used in residential, commercial, and industrial buildings. These devices are called “building energy management systems” in certain localities. A building is considered to have smart qualities when automation, sensors, and other remote elements are used to improve the effectiveness of building administration, the level of tenant contentment, and the expenses associated with building maintenance.

The Internet of Things technology can improve and optimize computational models related to electrical networks. This is made possible by the combination of user data and the prices charged by energy providers. The optimization made available by the Internet of Things could result in improvements to computational models; nevertheless, it is also possible that modifications will result in performance issues and network noise. The focus is investigating statistical aggregation’s complexities, subtleties, speed, and correctness (Succetti et al., 2023). The information that is provided in this article was gathered from several sources, including customers, suppliers, and smart meters. Difficulties are caused by altered data propagated throughout the network due to transmission, quantification, and essential consumption measurement defects. These difficulties are caused by the inability to measure essential consumption accurately.

Because NS-3 has excellent simulation coverage, BPLC can satisfactorily fulfill the SG’s bandwidth requirements. Through NS-3 simulations, the system examines a wide range of components to demonstrate the capacity of a line to carry both power and data. The system’s purpose is to carry out actions that have been collected in the past few times. Achieving substation output matching for an application-layer transmission rate is possible when UDP/IP is utilized as a support mechanism. It is important to remember that certain variables, such as the coupling, surroundings, and cable age, cannot be recreated under any circumstances. The programmable logic controller (PLC) technology (Abideen et al., 2023) was made. This innovation was made possible by the technology.

When considered within the context of the CR-AMI network, Green-RPL emerges as an efficient protocol regarding energy consumption and loss-routing abilities (Mazhar et al., 2023a). The prioritization of packet routing is affected by the estimated virtual distance (EVD), and the protocol ensures the node transfer that consumes the least energy by picking the economically cost-effective technique. Throughout these activities, the requirements for utilities that the smart grid and secondary consumers impose are efficiently met.

The literature discusses smart grids, sustainable cities, and related technologies but has a few notable gaps. It primarily focuses on technological and infrastructural aspects, lacking in-depth social, cultural, and economic exploration. While cybersecurity is acknowledged, a more detailed analysis of specific challenges is needed. The role of government policies and human-centric design principles should be emphasized. Additionally, practical examples, environmental impact assessments, and studies on public perception would enhance the overall understanding. Addressing ethical considerations and providing a more nuanced view of renewable energy integration would contribute to a more comprehensive examination of the subject. Incorporating these aspects would provide a more holistic understanding of the challenges and opportunities associated with smart grids and sustainable city development.

## Methods and Materials

### Research questions

**Figure 5:**
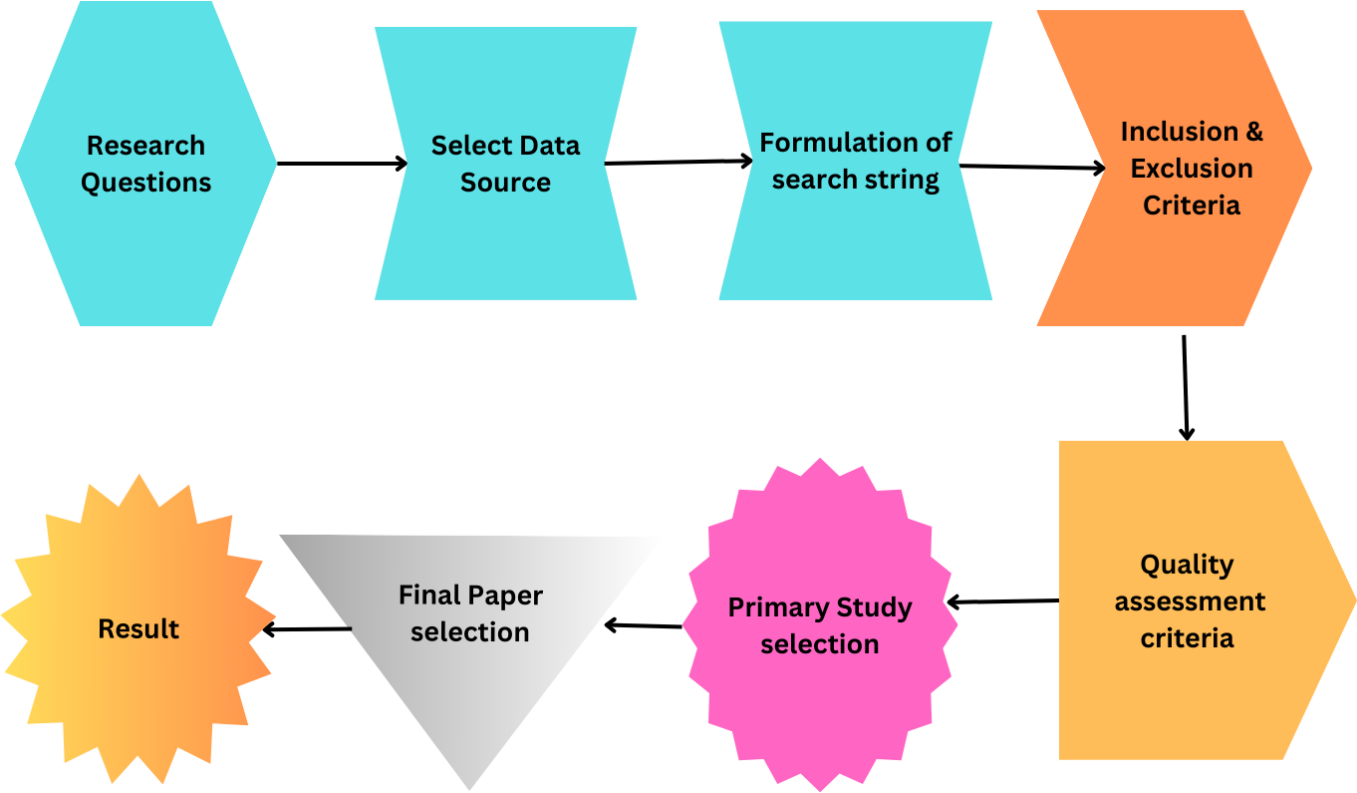
Proposed methodology.

The primary objective of this study is to conduct an SLR that identifies, analyzes, and summarizes empirical evidence related to using smart grid security using AI and big data. The review focuses on different types of attacks on the smart grid. It also focuses on the solution of these issues by using AI and big data techniques. The research questions and the motivation behind each question have been formulated to guide the review process to achieve this goal. Figure 5 illustrates the proposed methodology and Table 4 illustrates the query results from data sources.

**Table 4:** Query results from data sources.

Library	Initial	Title and keyword	Abstract	Full text
ACM	200	100	50	20
IEEE	150	80	40	18
Science Direct	80	65	35	22
Springer	100	50	45	30
Wiley	50	30	25	22
Results	580	325	195	112

1-: What are the different types of attacks on the smart grid?
2-: What are the security challenges of smart grids using AI and big data methods?
3-: What is the role of big data in healthcare?
4-: What are possible solutions to overcome security challenges of smart grid?

### Select data sources

**Figure 6:**
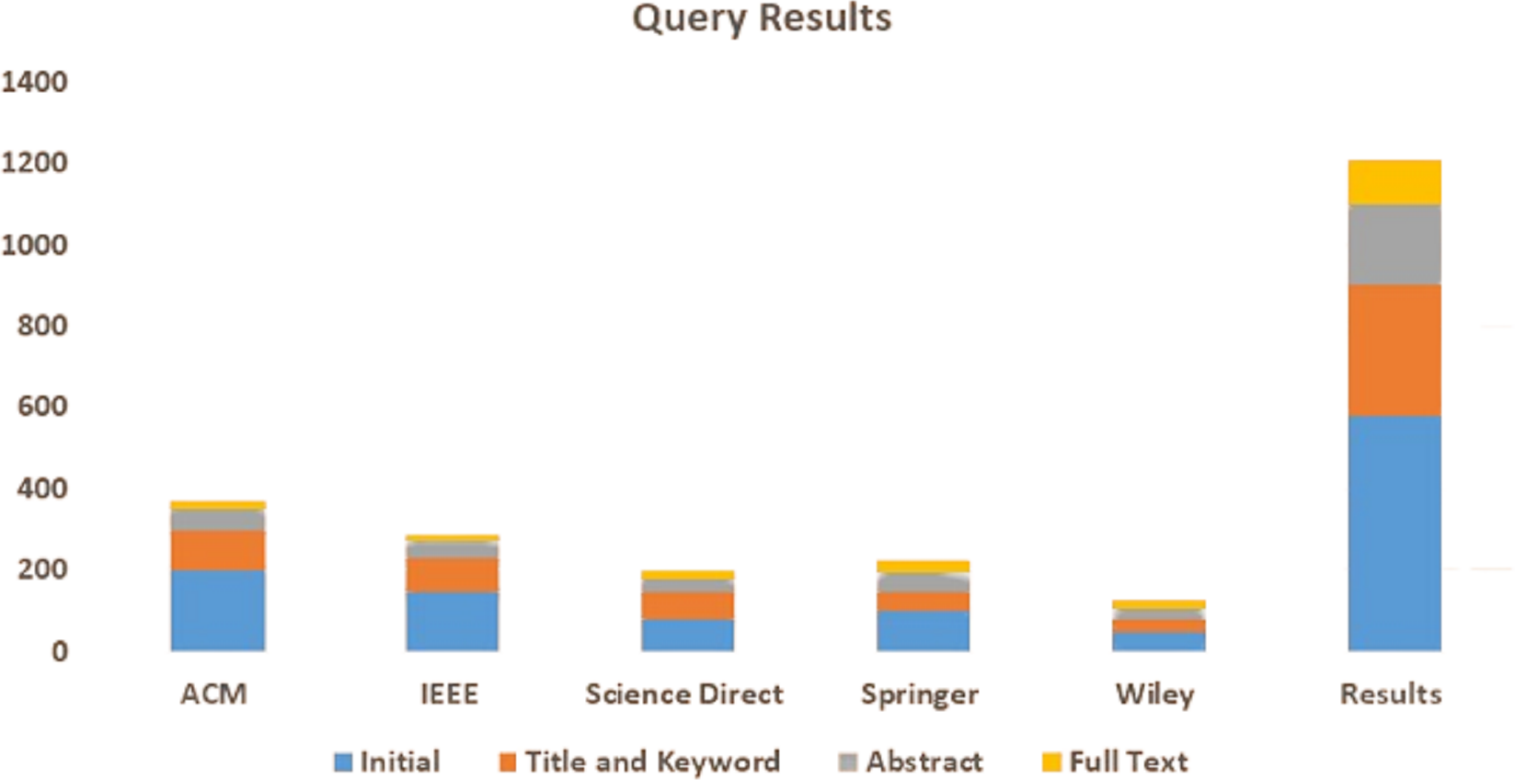
Query representation.

Data sources are the libraries or repositories from where the research studies should be retrieved. Five digital libraries, IEEE, Springer, Science Direct, ACM, and Wiley, have been chosen to extract the primary data, as depicted in Fig. 6. Documents are searched to identify the prior studies. There are various options available to search each digital library for pertinent information. To find the most relevant literature, the search strategy is modified to satisfy the respective needs. Table 5 presents the query results from data sources.

**Table 5:** Search string formulation.

Keyword	Synonym/Alternative word
Smart grid	(“Smart meter” OR “Smart system”)
AI	(“DL” OR “ML”)
Security	(“Privacy” OR “Protection ”)
Methods	(“Techniques” OR “Framework ”)

### Formulate search string

**Figure 7:**
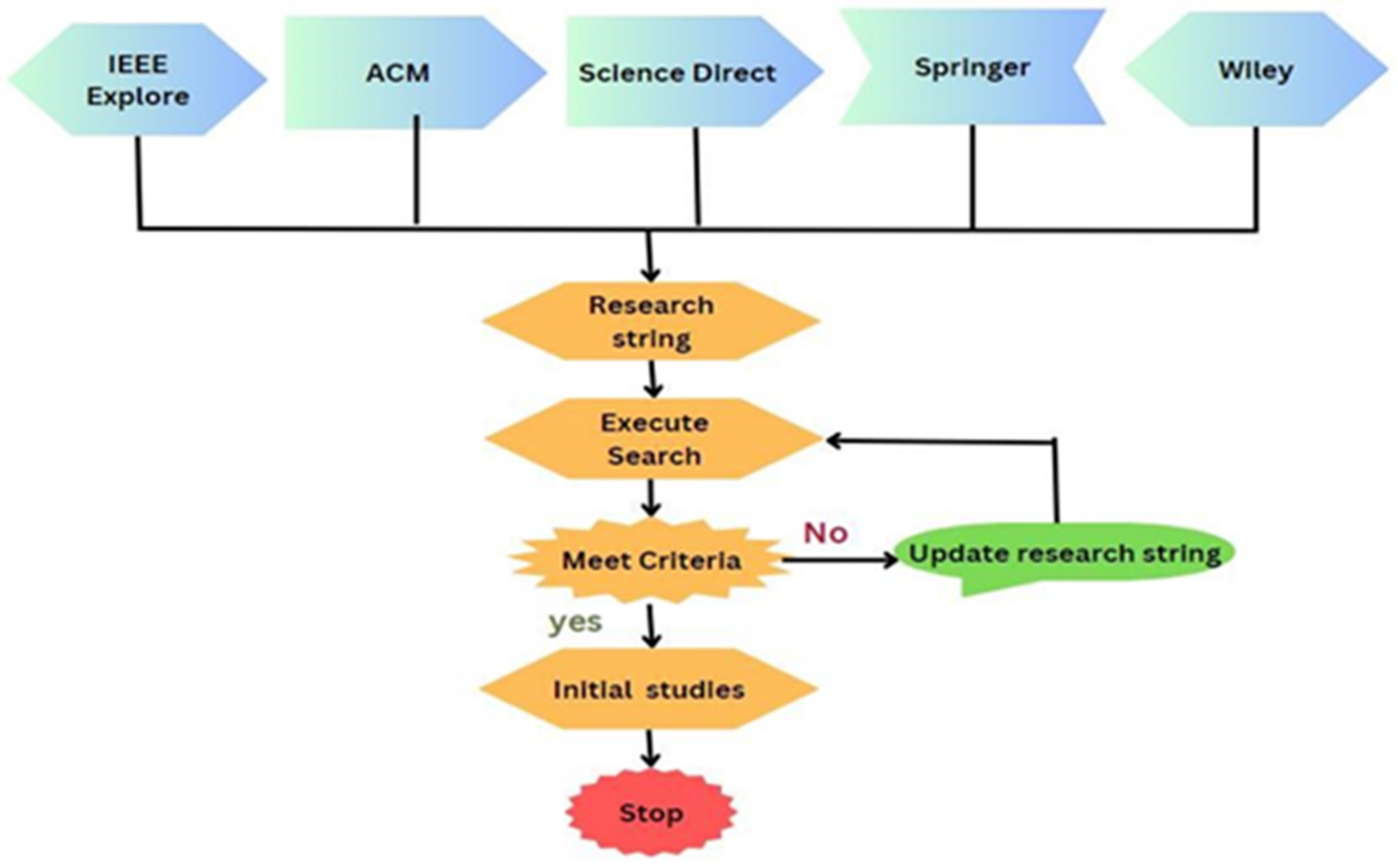
Formulation of the search string.

A search string is a carefully crafted combination of keywords and search operators used to identify relevant studies that address the research question or topic of the review. This step focuses on specific keywords and synonyms from the identified research questions to create the search string. These keywords are combined using the ‘AND’, ‘OR’ conditions in the order listed to complete the following search string. Figure 7 and Table 6 illustrate the process of formulating a search string.

**Table 6:** Quality assessment criteria.

Sr. No	QA questions
C1	Are attacks on the smart grid clearly defined in the study?
C2	Does the current research on AI and big data provide enough information?
C3	Does the use of countermeasures provide enough information?
C4	Are the challenges and risks of applying AI in a smart grid clearly defined?
C5	Are the possible solutions to critical challenges related to AI and big data clearly defined?

### Define inclusion and exclusion criteria

Inclusion criteria in an SLR refer to the predefined rules used to determine which studies would be included in the review. In this review, the following inclusion criteria will be considered:

• Studies must have been published in English from 2014 to 2023. The subject of the study should be centered on smart grid security utilized in the domain of security and AI.
• The investigations undertaken in the study should relate to the attacks on smart grids.
• The investigations undertaken in the study should relate to the solution of attacks on smart grids.
• The scope of selected articles should be confined to publications in reputable journals, conferences, or books.

The following categories of studies have been designated for exclusion:

• Those published before 2014. Studies that lack empirical analysis results
• Exclusion criteria in an SLR refer to predesigned conditions to determine which studies will be excluded from the review.
• Those whose primary focus is not on smart grid security and AI.

### Define quality assessment criteria

Quality assessment criteria in an SLR refer to the predefined standards or guidelines used to assess the included studies’ quality, reliability, and validity. Defining quality assessment criteria ensures that the selected primary studies offer sufficient details to effectively analyze the identified research question. In this step, a standard is defined against each research question. Each quality assessment criterion is denoted by C and its respective number, as shown in Table 7.

**Table 7:** Final article selection.

Year	Final selection
2014	04
2015	03
2016	8
2017	7
2018	12
2019	21
2020	16
2021	13
2022	20
2023	8

### Primary study selection

**Figure 8:**
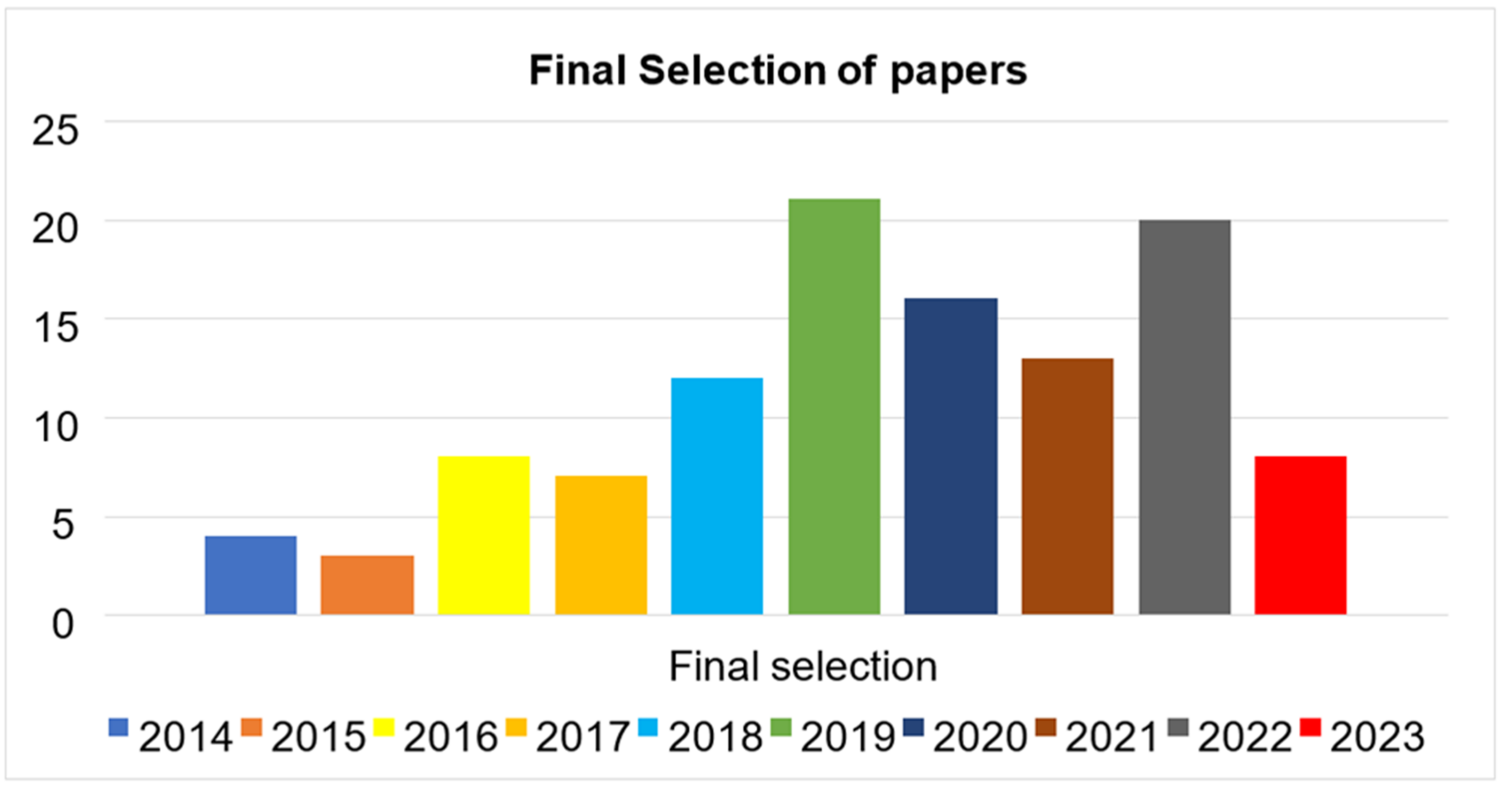
Final article selection.

**Figure 9:**
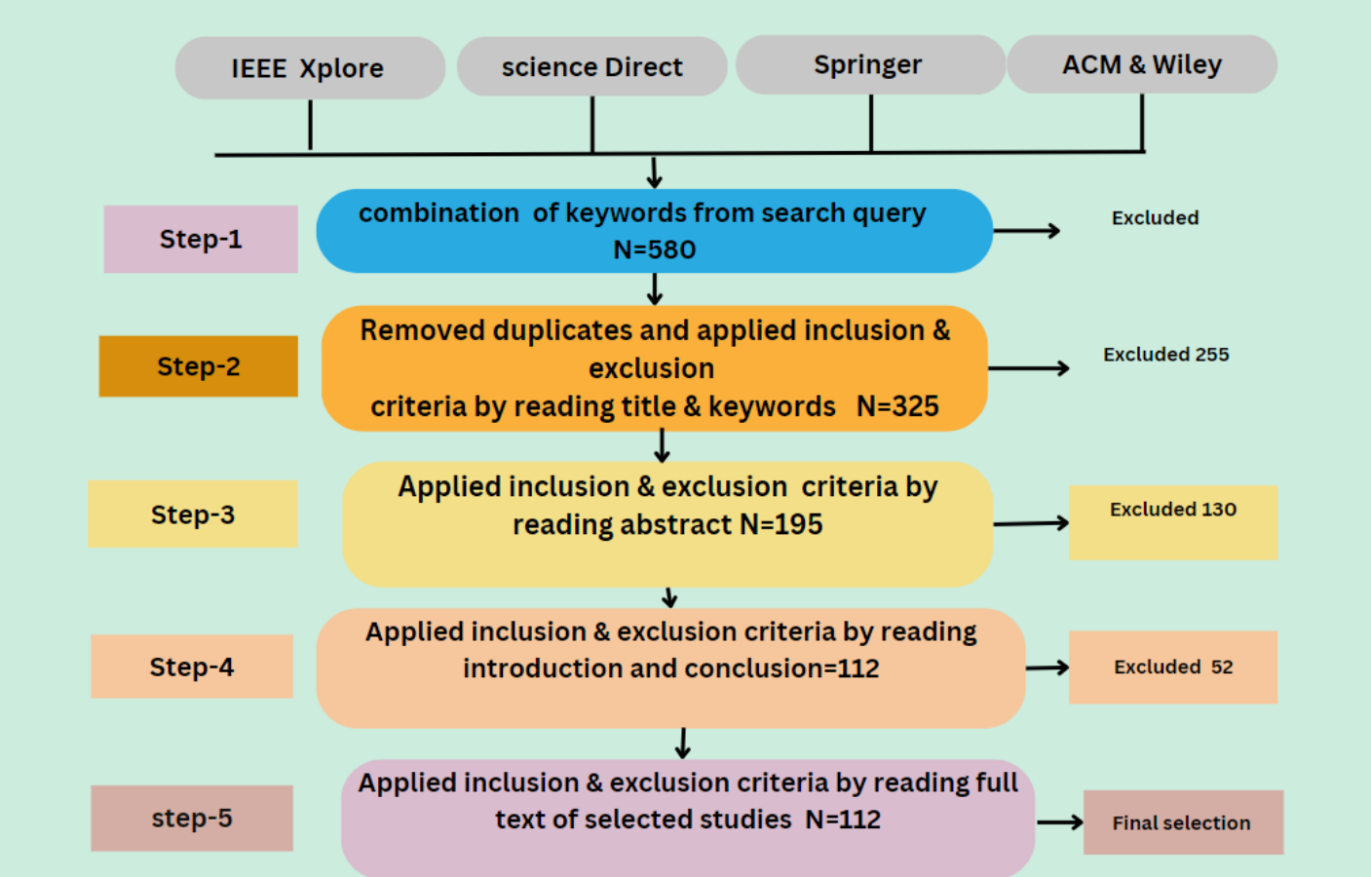
Prism diagram.

Primary studies refer to the individual articles or book sections that directly address the research questions or topic of the review. This review has selected prior studies using the tollgate approach, a structured methodology of five phases (Kitchenham & Charters, 2007). This approach was instrumental in carefully curating 49 primary studies, considering the specified quality criteria for prior studies. The primary study selection is illustrated in Table 8, and the overall process is presented in Fig. 8. The prism diagram is shown in Fig. 9.

**Table 8:** Summary of existing work related to BC, ML, and SG.

Ref	Major contribution	Technical resources
Cheng et al. (2023)	Evaluate the steps to construct a decentralized network to recharge electric vehicles using BC, AI, and SGs.	Predictive resources, distributed stretcher
Mololoth, Saguna & Åhlund (2023)	A decentralized architecture is proposed to facilitate electricity trading among electric vehicles (EVs) connected to the grid.	Predictive price, computer-generated deals, and the use of the Hyper ledger architecture
Jamil et al. (2021)	This explains how BC and ML can be utilized in a decentralized marketplace between peers in SGs to exchange renewable energy.	A prediction model that was achieved by the utilization of PBFT, LSTM, Hyperledger, and smart contracts
Khalil et al. (2022)	This is a comprehensive description of how the British Columbia consortium could be utilized by developing energy companies to create intelligent charging infrastructure for electric vehicles.	Memory-restricted neighbourhood searches are made possible by smart contracts and algorithms developed by third parties (LNSM).
Ashfaq et al. (2022)	Because of this, implementing a trading system for energy powered by AI and distributed ledgers is encouraged.	Smart contracts based on the k-nearest neighbour algorithm

## Results

### Attack in smart grid

Hackers get access and control *via* scanning, monitoring, performing maintenance, and modifying equipment. The observation stage of an attack is when the attacker learns as much as possible about the target. Finding the system’s weaknesses is the second stage. Through these tasks, students will learn about maintaining and detecting problems with the open port operating system (Ghiasi et al., 2023). By losing system control, they try to win during goal manipulation. Once access has been provided to the appropriate administrative levels, the transfer process is complete; at this point, it must be granted permanently. They do this by secretly installing software on the target system that enables them to return whenever they want without being noticed. Due to this security failure, attackers must follow SG’s security policies. They use different strategies at each level to breach the SG’s defenses. Therefore, we can use these methods to categorize cyberattacks (Goudarzi et al., 2022). It demonstrates the several kinds of attacks that may take place throughout the define stage. Attacks and bad things have happened everywhere. Attacks like traffic analysis and social engineering are used in military missions. In social engineering, relationships with other people and people skills come before technical knowledge. An attacker will use trickery and seductive language to gain a victim’s trust and acquire sensitive information, such as login passwords. For instance, SE has several.

**Figure 10:**
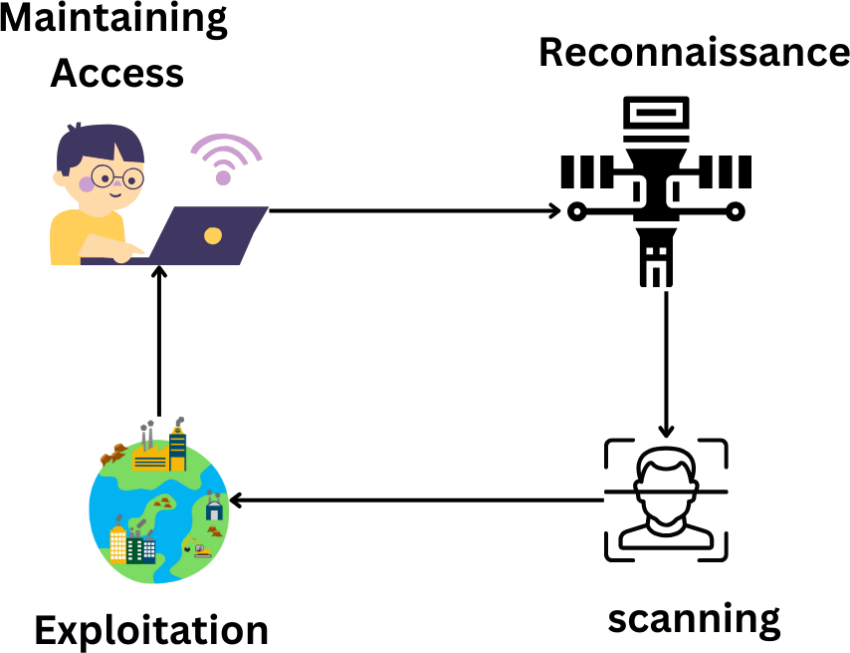
Hackers follow the attacking cycle to get control over a system (El Mrabet et al., 2018).

Passwords and phishing attempts. Using network traffic monitoring, managers can determine which servers and devices connect to an incoming attack. Most computer systems are vulnerable to compromise through social engineering and traffic analysis. Figure 10 explains the attacking cycle.

### Scanning

**Figure 11:**
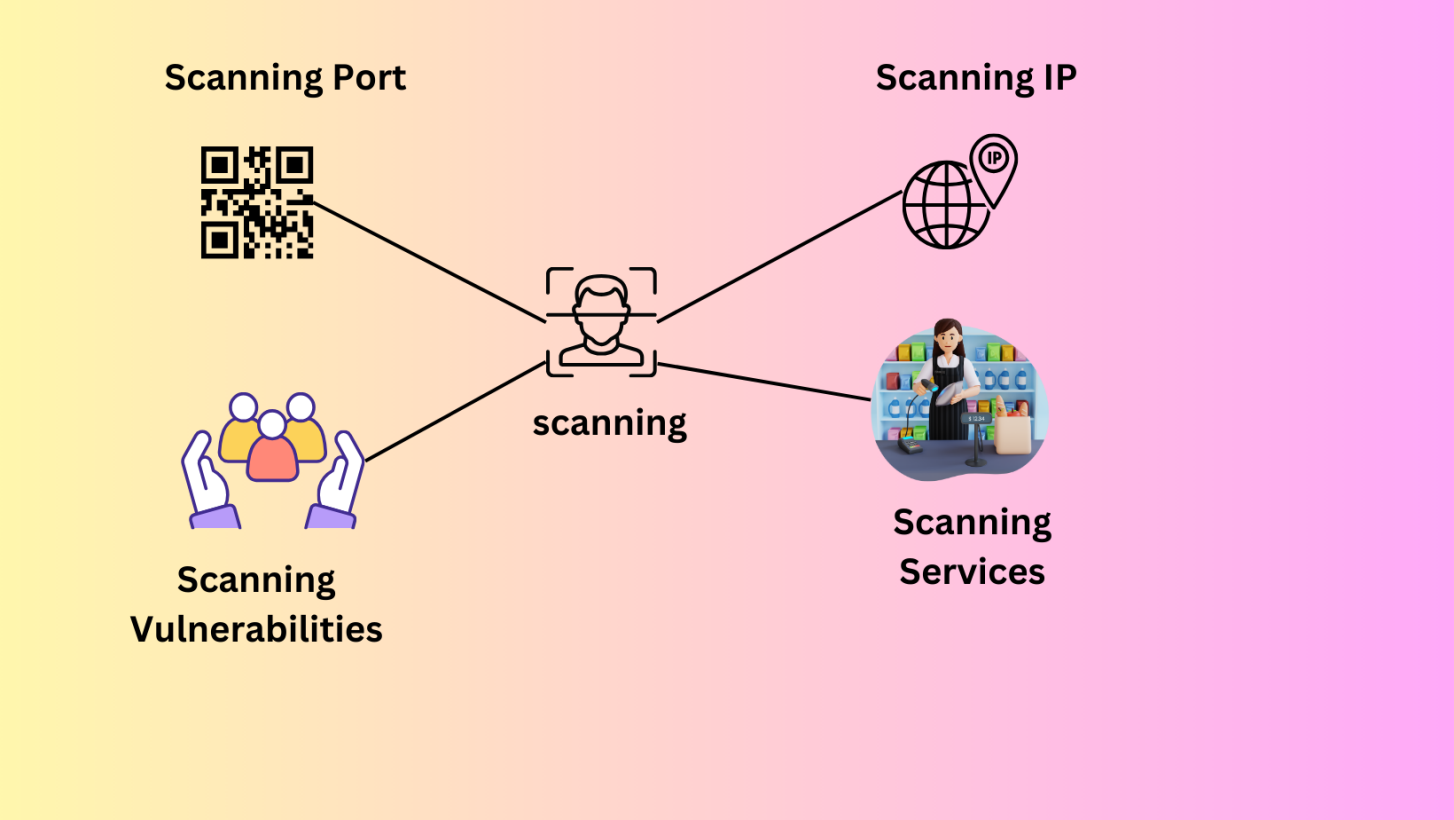
Scanning process (Zhang, Liu & Wu, 2021).

The next stage is a scanning attack to determine which hosts and PCs are still running. When scanning, IP addresses, ports, utilities, and security flaws must be considered (Hasan et al., 2022a). An attacker usually performs an IP scan of the hosts connected to a network using newly acquired IP addresses when they first get access. They then travel a little further to each port to consider their options. Every host network that has been found runs a scan. The next step taken by the attacker is a service scan to identify the kind of device or service that is listening on each open port (Hasan et al., 2022a). The vulnerability scanning phase follows, looking for weaknesses, goals, and weak points in each service system on the targeted devices. Industrial protocols that are vulnerable to scan attacks include Modbus and DNP3. To stop hackers from breaking into the communication system *via* Modbus network scanning, TCP/Modbus was developed. Every machine on the network receives a message that the attacker sends that seems safe. This message is sent to such devices to steal their data. Mods scan a well-known SCADA Modbus network scanner that can find and open TCP/Modbus connections, system IP addresses, and slave IDs. Figure 11 explains the scanning process (Zhang, Liu & Wu, 2021).

### Exploitation

**Figure 12:**
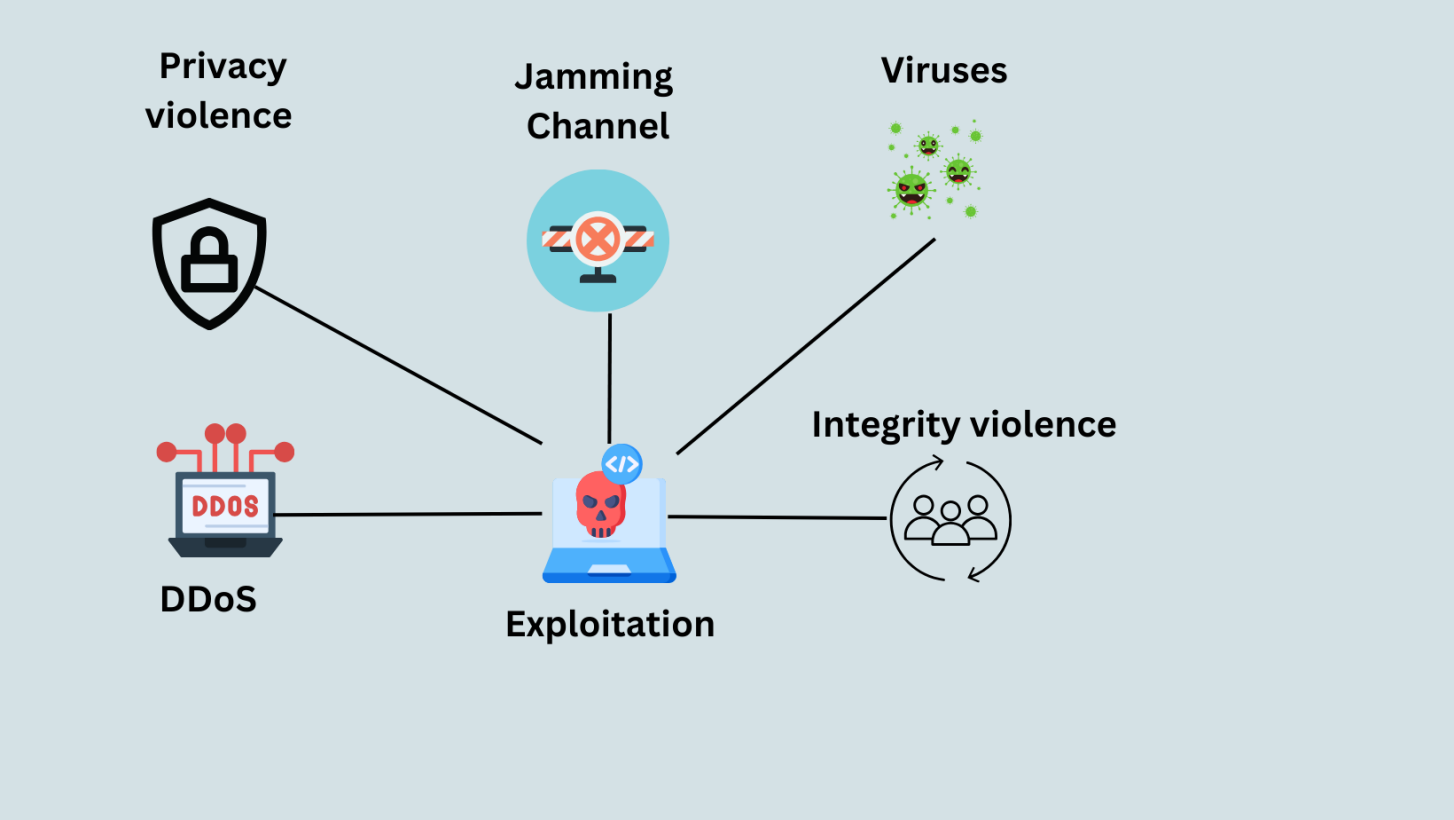
Exploitation process (Mohammadpourfard et al., 2021).

Attack activities use the SG system’s components in the subsequent “extraction” phase to take over control and locate weak points (Musleh, Yao & Muyeen, 2019). Such attacks include man-in-the-middle, denial of service, and replay assaults. Other examples include privacy violations, channel jamming, integrity breaches, viruses, worms, and Trojan horses that compromise human-machine interfaces. Malicious software created to transmit from one computer to another is a virus in the smart grid (Hussain et al., 2022). A “worm” is a piece of software that can duplicate itself. It makes copies of itself and spreads them to other devices and computers (Mollah et al., 2020). Trojan horses are harmful programs that give the impression of helping the computer they are installed on. However, in this case, it runs destructive code. This kind of malicious software is used by criminals to infect target systems with viruses and worms (Mohammadpourfard et al., 2021). Figure 12 explains the Exploitation process.

### Maintaining access

**Figure 13:**
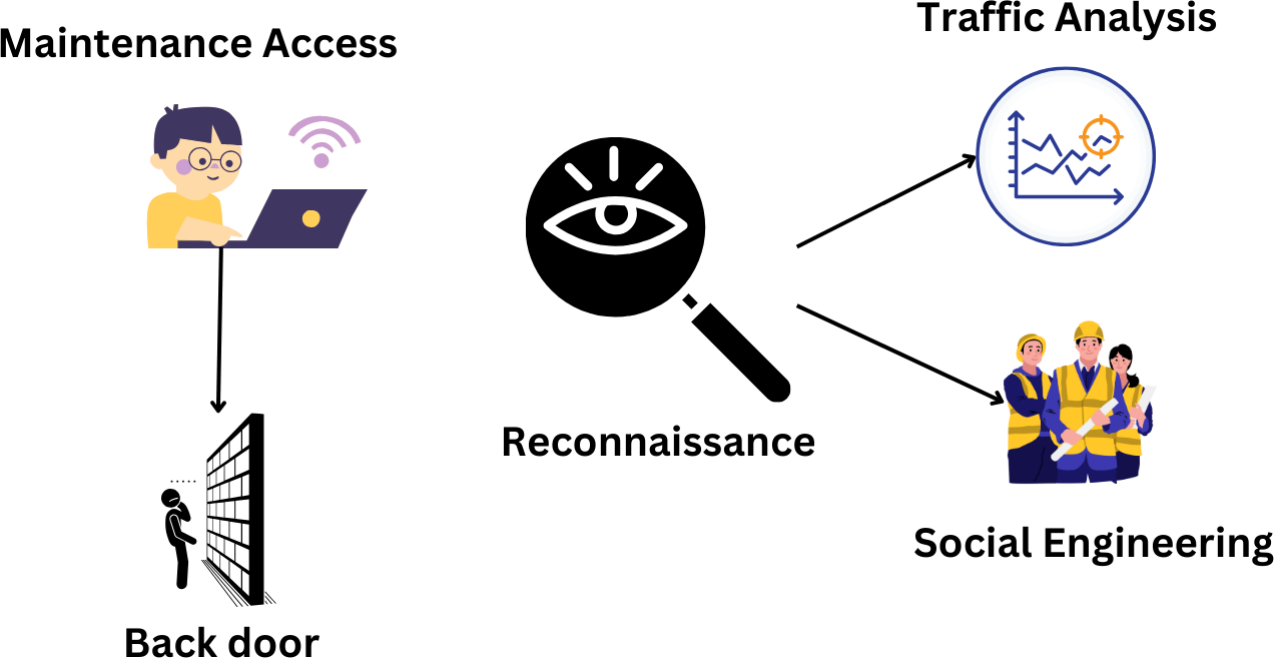
Maintaining access process (El Mrabet et al., 2018).

In the last stage of an attack, the attacker uses a specific attack technique, like a backdoor, virus, or Trojan horse, to get unrestricted access to the target system. Installing a backdoor or other undetected malware enables quick and easy access to the target (Abideen et al., 2023). Let’s say the enemy successfully surrounds and controls the SCADA server. They might start a series of attacks against it in this situation, which would be dreadful for the electrical grid (Wasel & Maan, 2020). An IT network’s four most important components are availability, honesty, accessibility, and privacy. They stand out in the SG for their transparency, integrity, openness, and privacy. As a result, attacks that could decrease the usefulness of smart grid networks are taken very seriously. Privacy threats, however, are generally not taken seriously by people. Each attack has a chance of happening and a level of risk. They are complex and challenging to use, albeit (Kolomvatsos, Anagnostopoulos & Hadjiefthymiades, 2015). Because of this, even though these viruses are dangerous, they do not regularly spread (Demir & Suri, 2017). Fig. 13 explains the maintenance access process.

### Impact of the cyber attack

A significant financial hurdle for the SG is integrating a substantial proportion of renewable energy into the system. Current and future transactions are available to dealers in the energy market. A day market focused on forecasting and optimizing load at the lowest cost. At each bus stop, the optimization problem determines the local maximum power price (Chen et al., 2021). This is significant because FDI CAs on the day-ahead market might affect load predictions. The real-time market, in contrast, continuously tracks the energy consumption and production rates for each route.

The power capacity of each line can be determined using real-time LMP, which shows the congestion pattern. This suggests that FDI state calculation significantly impacts the current market, as mentioned briefly in Hasan et al. (2022a). Attacks against the FDI have significantly harmed technological and material infrastructure. A stable steady-state smart grid is typically present during FDI attacks and has immediate effects. Attacks by FDI on steady-state stability have significantly impacted smart grid voltage control and energy management. Although FDI can affect how the SG regulates frequency, the objective is to keep the rotor angle constant. Every assault took place inside the SG defense network (Majidi, Hadayeghparast & Karimipour, 2022). Table 9 illustrates previous BC, ML, and smart grid work summaries.

**Table 9:** Artificial intelligence (AI) and cybersecurity.

How AI can help in cybersecurity	References
Detection by automation	Falco et al. (2018)
Errors in quick identification	Lin, Liu & Wang (2019)
Secure authentication	Zhang & Yan (2019)
Quicker response times	Barati (2019)
Error-free cybersecurity	Hu, Yan & Wang (2019)

### Security aware of SG infrastructures in the era of big data and artificial intelligence

**Figure 14:**
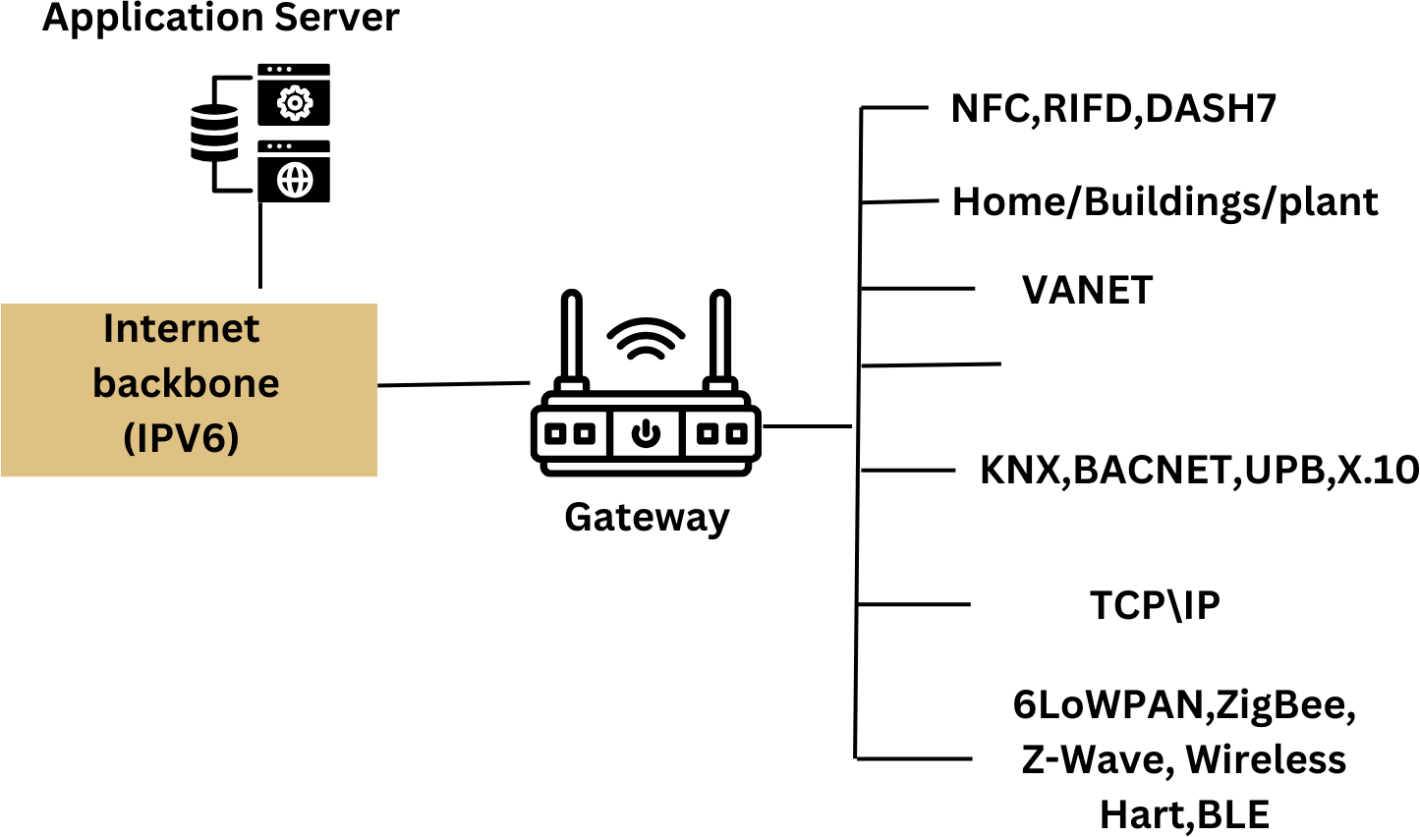
Information and communication technologies (Meddeb, 2016).

Smart meters are particularly vulnerable to SG flaws because they constantly change as electricity is generated and used. This depends on where the meters are and the encryption key used to protect the data from the energy analysis tools (Avelar et al., 2015). The use of digital technologies in the electricity grid’s physical architecture is called the “smart grid.” Because of this, it is simple for utility providers and customers to develop solutions that guarantee the reliability and continuity of the electrical supply while ensuring optimal performance because the system runs independently. Some SCADA systems and components are no longer in use because they have been around for a while. Some were created before the widespread understanding of cybersecurity best practices. Because SCADA systems are not Internet-connected, their manufacturers may claim that cybersecurity is not essential. However, SCADA systems expanded as the internet developed, and many were built without security. Modern, safer technology might easily replace the traditional system, which is frequently delayed owing to cost. The SCADA network is required to protect the larger plant’s control system against attack. The SCADA network and the company network could each have a second firewall with more restrictive rules placed between them. The implementation of security measures, analysis of log files, and distribution of updates would be possible by authorized service engineers to assist and monitor security. The communication network needs of the main SG applications for local air networks, near air networks (NAN), and global air networks are examined in critical communications studies. The most crucial security services to look at were listed by the author. Different technologies are present in the Internet of Things. Examples include Bluetooth, ZigBee, Wi-Fi, NB-IoT, and LTE. The many ICTs that can be used in a power grid are shown in Fig. 14.

### The enormous potential of big data

**Figure 15:**
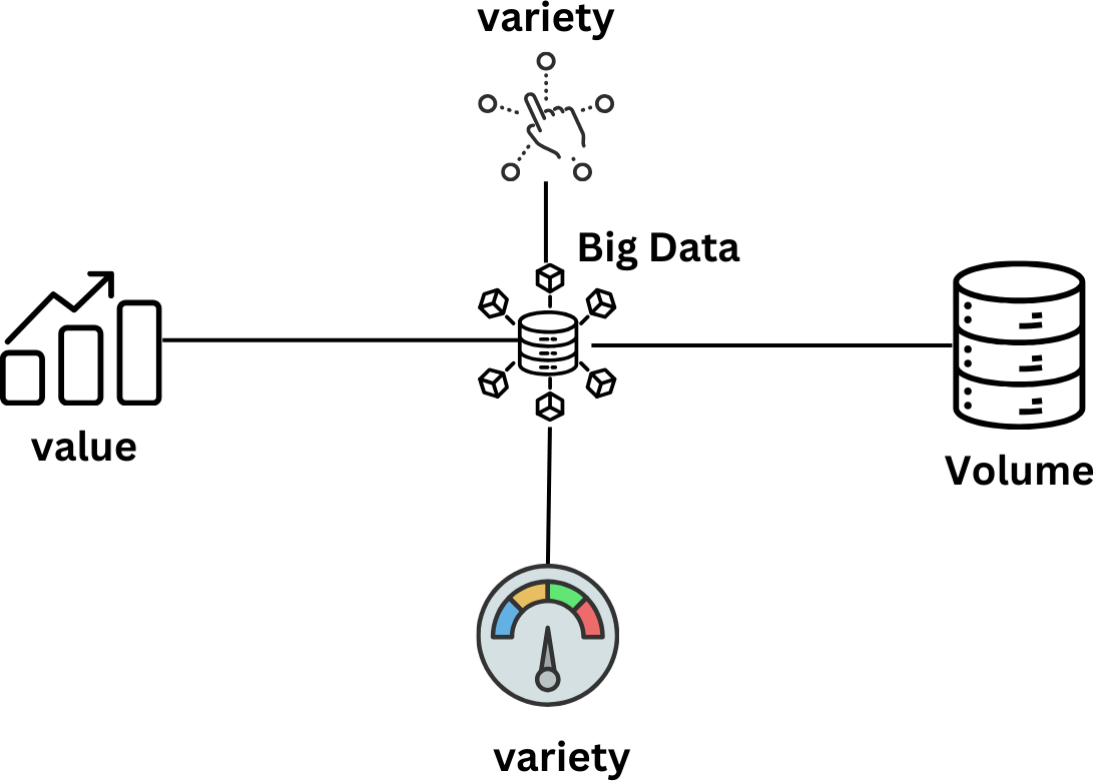
The properties of big data are reflected by 5Vs (Tsai et al., 2015).

On May 6, 2017, The Economist announced that data had overtaken heavy crude as the most valuable resource in the world. In the absence of a universally accepted definition, “big data” is defined as “a vast quantity of information that requires the use of tools other than those found in standard applications programmed to analyze it (Soundararajan et al., 2014). Due to the size of the database, it is challenging to gather data, store it, analyze it, keep it current, search it, send it, see it, update it, and protect it. Three main approaches—pooled data analysis, a meta-analysis of summary data, and federated data analysis—can be used to analyze synchronized information from various sources (Kolomvatsos, Anagnostopoulos & Hadjiefthymiades, 2015). Figure 15 explains that 5Vs reflect the properties of big data.

Due to knowledgeable algorithms, the SG can see the overall picture of these energy sources and needs in real-time or in advance. The smart grid might automatically modify the network’s energy flow using this information. As a result, areas with high energy needs are supplied with electricity, mostly from renewable sources. Producers of electricity are working on big data and open data at the same time. big data is the term used to describe the rapid growth of digital data (Tsai et al., 2015).

### Cybersecurity and artificial intelligence

**Figure 16:**
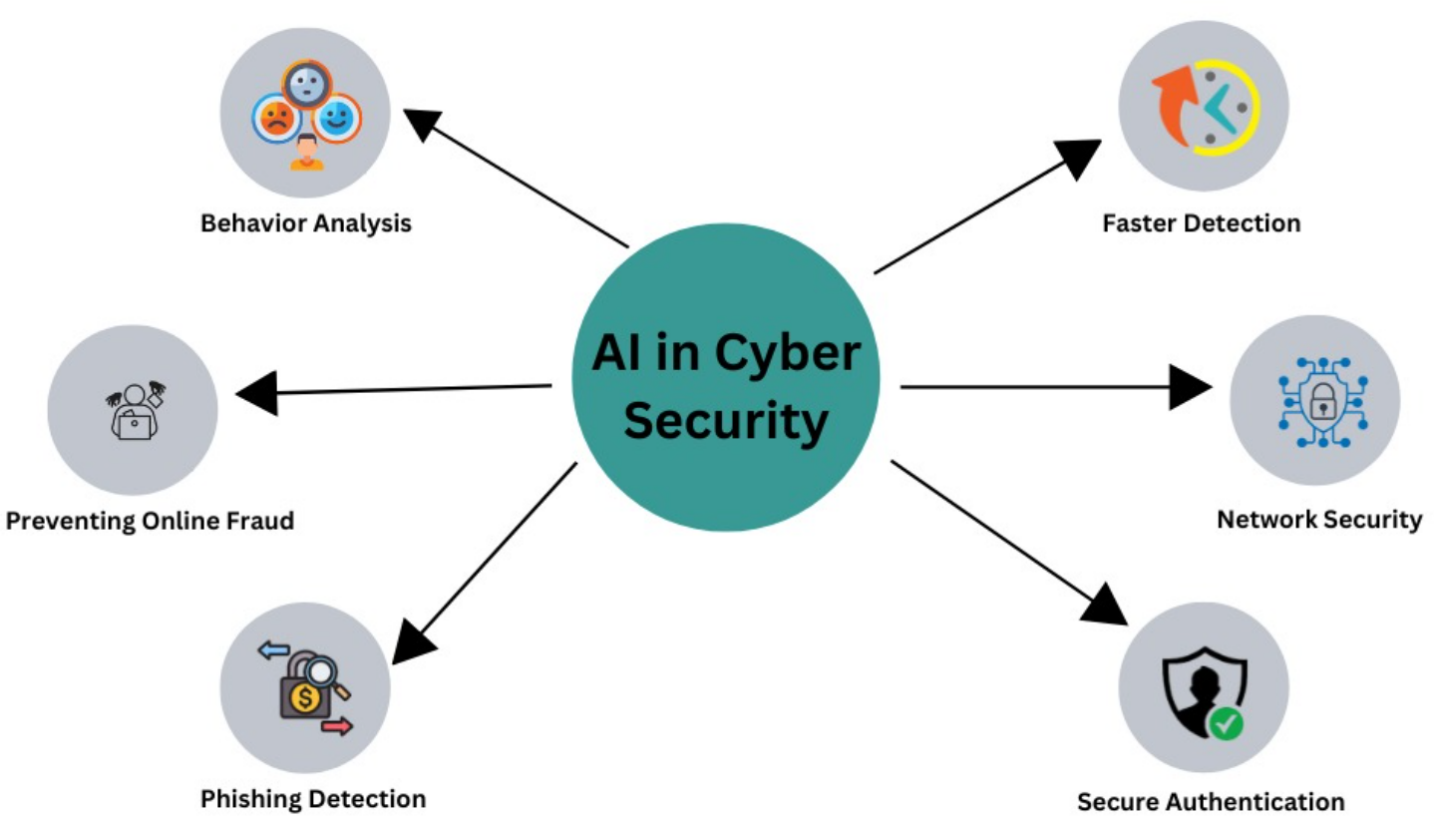
AI in cybersecurity.

The field of cybersecurity has many uses for AI (Avelar et al., 2015). Robot-assisted process automation, ML, and NLP are frequently used in the digitization of manufacturing processes (Chehri, Fofana & Yang, 2021). Consider the filtering system, which has been used since the early 2000s (Dada et al., 2019), as an example of how ML might be useful. It is clear that techniques have changed over time, and modern algorithms can make more complex choices. Recent AI developments have significantly improved smart grids’ digital security, enhancing defenses against various threats. Security privacy, business, and information technology are the five main uses of ML. Many people may be unaware of how widely AI is used. AI enables businesses to quickly identify risks, speeding up response times and ensuring they meet the best security standards. The energy sector must continue investing to remain ahead of cyberattacks, even while technologies like AI and 5G are ready to aid in problem-solving (Hassani et al., 2020). Deep learning systems are skilled at user monitoring, and AI is essential in detecting and preventing breaches in computer networks. Identities if needed. Figure 16 describes the relationship between AI and cybersecurity.

AI algorithms can detect anomalies such as accessed databases, frequent location changes, and unusual access times (Necip Kurt et al., 2018). ML, on the other hand, makes it easier to find data patterns that support automated learning (Ahmed et al., 2018). By utilizing their understanding of cyber threats, smart grid users can quickly address problems. While current security systems are excellent at observing and preventing typical threats, they cannot keep up with the changing requirements for cybersecurity. Zero-day vulnerabilities, which are used by extremely slow cyberattacks, cannot be mitigated by them. Examining datasets and finding hidden security flaws requires a more flexible methodology (Li et al., 2019b). Through adaptive baseline behavior models, machine learning has successfully identified novel dangers. The security landscape could significantly change if machine intelligence and predictive analytics are combined with known and unknown datasets (Singh, Yassine & Benlamri, 2018). A summary of how AI can improve cyber security measures is shown in Table 10.

**Table 10:** CORAS method for security risk analysis.

Attacks references	Attacks references
Attacks using switch	Wei, Wan & He (2019)
DOS	Wood, Bagchi & Hussain (2017)
Detection of fraud	Korba & Karabadji (2019)
Detection of cyber threats	Yan, Tang & He (2016)
Integrity of data	An et al. (2019)
Dropping of replay packets	Yadav et al. (2016)
Data injection attacks with dynamic load altering	Velusamy & Pugalendhi (2017)
Viruses and malware (Malware)	Zhou et al. (2019)
Vulnerability assessment	Cao et al. (2020)
Detection of anomalies	Hong et al. (2020)
Attacks using switch	Hasan, Shetty & Ullah (2019)
DOS	Weisha, Jinguang & Jiazhong (2018)

### Big data and health awareness

Most big data that positively affects health may be seen in three areas: illness prevention, identifying important. Health risk factors and enhancing healthcare interventions (Fanta, Pretorius & Nunes, 2021; Sheth et al., 2017; Aflaki et al., 2021). By giving detailed information about each person’s medical history, big data aims to boost the use of electronic health records (El Samad et al., 2022). Efficiency, rapid diagnosis, and individualized therapy may be where the advantages of technology, particularly the capacity to store and transmit large amounts of clinical data, are most readily apparent (Shams et al., 2022). Due to recent biotechnological developments, the “individual” can now be treated in all of their individuality” (Gligorijević, Malod-Dognin & Pržulj, 2016). This has critical medical advantages (Parimi & Chakraborty, 2020). A detailed analysis using many machine learning algorithms and the delivery of consistent, appropriate, safe, and flexible solutions are used.

**Figure 17:**
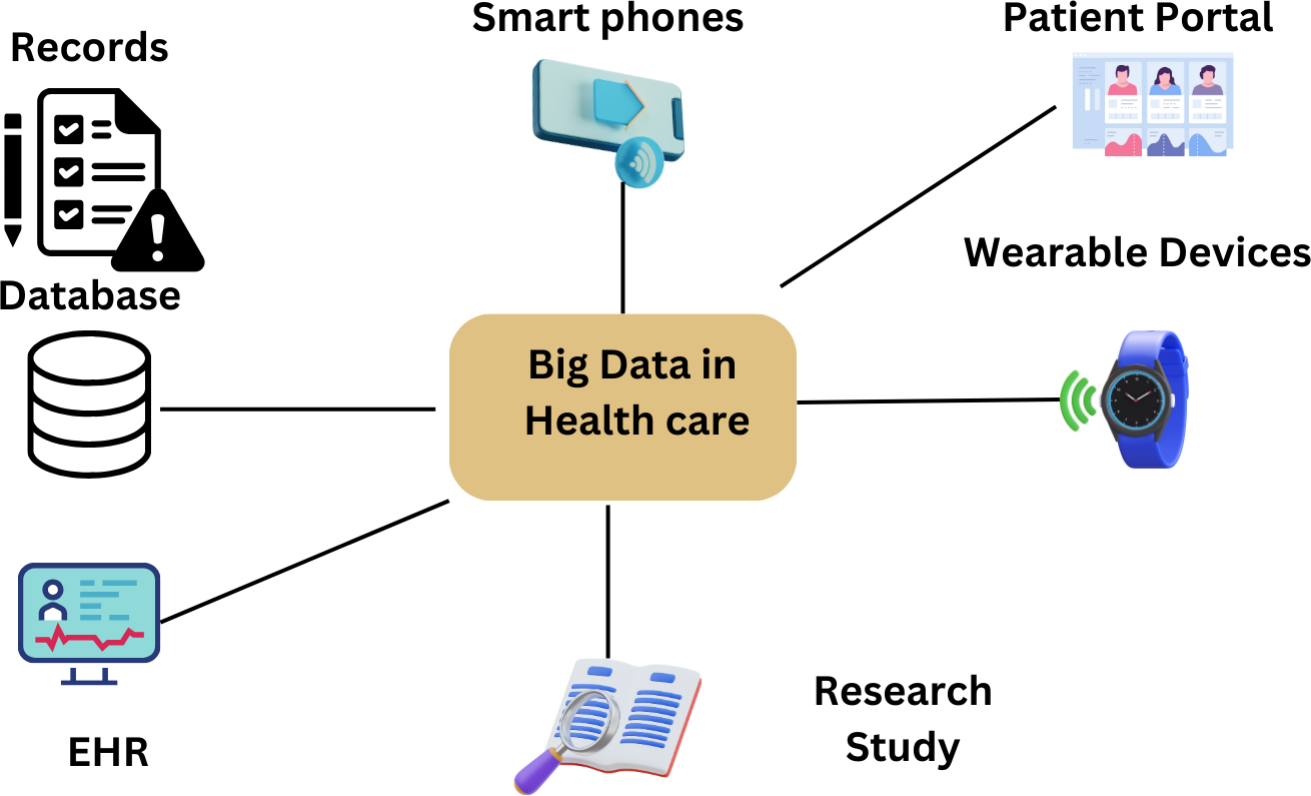
Big data in health (Radanliev et al., 2020).

This is more patient-centered and effective. Predictions made with BDA technology speed up the reporting of at-risk patients, resulting in more effective and efficient care and better overall health outcomes (Radanliev et al., 2020). Following population movements and trends is crucial for early diagnosis and personalized health care, made possible by the data’s diversity, volume, and velocity. All research in this field in 2021 has shown that big data management is crucial to raising the standard of healthcare and patient outcomes (Dicuonzo et al., 2022). AI-based diagnosis-based techniques and algorithms might be used to find outbreaks before they spread. Several technologies could help control the SARS-CoV-2 virus and the associated sickness. COVID-19. This would increase the efficiency of medical resources and decrease the possibility of a pandemic starting in a single nation. AI, Industry 4.0, the Internet of Things, the Internet of Medical Things big data (BD), virtual reality, drone technology, autonomous robots, 5G, and blockchain have all contributed to the control of COVID-19′s spread (Shamila, Vinuthna & Tyagi, 2019). Another often-used technology is wireless body area networks (WBANs). The new way of doing things may completely change how healthcare is provided and provide several patient advantages (Hossain et al., 2018). Figure 17 explains the use of big data in health.

**Figure 18:**
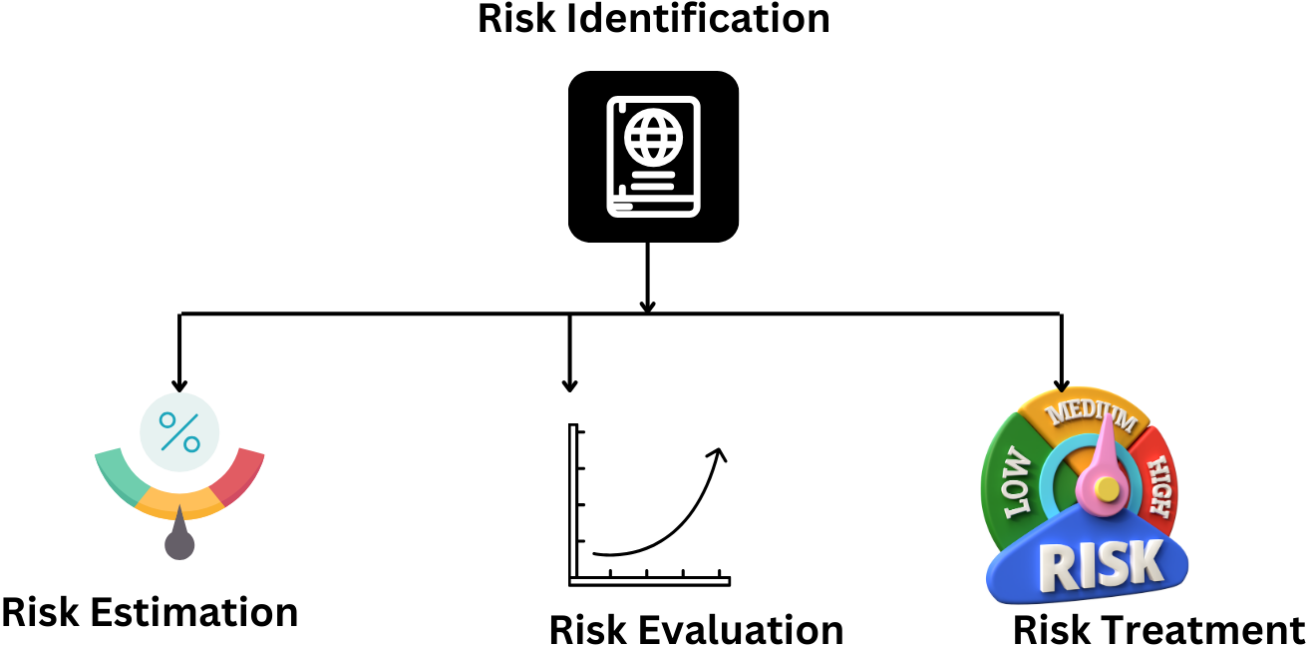
CORA’s method for security risk analysis.

**Figure 19:**
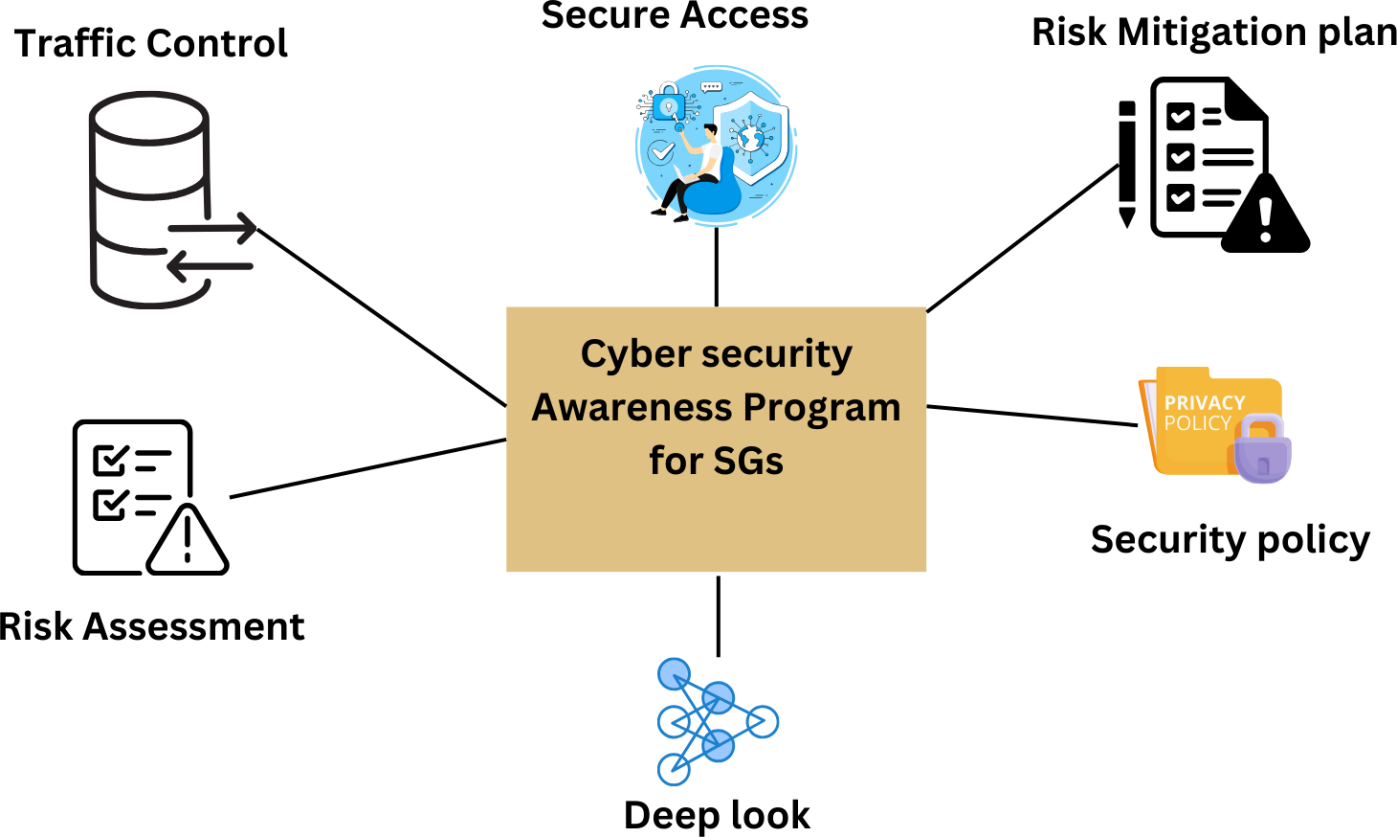
Mitigating the risk of cyber-attacks on smart grid systems (Rice & AlMajali, 2014).

By downloading apps directly to their smartphones and tablets, patients can keep tabs on their health and report on it. This is possible given how mobile healthcare has developed from digital healthcare (Fanta, Pretorius & Nunes, 2021). Therefore, IoT facilitates faster and more accurate patient diagnosis and healthcare delivery, especially in rural areas with no medical experts (Sheth et al., 2017). In the present SARS-CoV-2 pandemic, technology must be used to prevent and treat COVID-19 infections. Smart tags with monitoring and data scanning capabilities, other wearable devices that can detect essential parameters and forward emergency calls in the event of a problem, and a Real-Time Location System, a satellite-based system, are examples of wearable devices with sensors for monitoring vital signs. Most of these devices are designed for people with circulatory diseases and diabetes (Sarosh et al., 2021). Combining various big data sources and applying them cleverly and efficiently might help health professionals do several activities either alone or in groups in precision medicine, predictive medicine, and preventive medicine (Dicuonzo et al., 2021). It is claimed that digital technology can help the healthcare sector transition to a circular economy. These techniques can facilitate the collection, recycling, repair, and disposal of traditional medical devices, especially IoT.

### Deep learning-based cybersecurity techniques in smart grids

Deep learning models can be used when conventional techniques fall short because of the number of dimensions wrath (Poggio et al., 2017). Deep learning models contain advanced training tools designed to extract useful features. The problem of SG cybersecurity has been addressed using various deep-learning techniques. Two convolutional layers, two pooling layers, one fully convolutional, a hidden layer, and an output layer are the layers that make up this kind of network. However, many deep learning algorithms have been used to identify cyberattacks on smart grids, including deep neural networks, recurrent neural networks, and artificial neural networks. A Kaltman filter and a recurrent neural network may be used to identify FDIAs. The dynamic threshold is investigated to identify an FDI attack. This clearly shows how and where to determine FDIA utilizing the input and output signals of a power-togas and gas-fired generation facility scheduler. In addition, a hybrid neural network can locate FDIA without labelling the training set of data (Sawas, Khani & Farag, 2020). Also, the authors could recognize cyberattacks specifically directed at IEC 61850 communication protocols using deep learning techniques. The work has advanced this field on frameworks for energy theft detection, the Parlier algorithm, and intelligent grid energy privacy protection using convolutional neural networks (Albarakati et al., 2019). A security system created to protect an IEEE 1815.1-compliant power grid was presented. To find anomalies and confirm the viability of the proposed method, a range of attacks, including malware, FDI, and DR, are tested using a deep learning algorithm trained on a bidirectional recurrent neural network. A GAN-based intrusion detection system called MENSA was created to identify and categorize attacks on Modbus and Distributed Network Protocol 3. He et al. (2022) developed a DL-based neural network model to calculate the bypass state and determine the root causes of transmission line congestion.To recognize false results, researchers also used ensemble-based DL (Abdulaal et al., 2022). Two deep learning models are trained with data using a decreasing window of observations. The ensemble-based detector uses the most accurate model to identify instances of incorrect data. He presents a DNN-based classification method for determining energy theft from smart grids. A Bayesian optimizer is used to adjust the hyperparameters to make it simpler to spot energy theft (Lepolesa, Achari & Cheng, 2022).

### Machine learning-based cybersecurity techniques in smart grids

Smart grids frequently use machine learning techniques to identify and stop cyberattacks. Our focus was on using machine learning to identify cyberattacks on smart meters, which are a significant factor in the high cost of electricity. The authors used ML techniques to predict future electricity costs. Datasets are pre-processed in machine learning, and features are extracted using methods like Joint Mutual Information Maximization Kernel Principal Component Analysis and principal component analysis (Khan et al., 2021). The model is then trained using algorithms for machine learning. The results are then produced using the trained ML model. FDI attacks on state estimation through the use of machine learning. Commonly, supervised and unsupervised classifier ensemble learning is used to lessen the impact of dimensionality reduction (Ashrafuzzaman et al., 2020). Principal component analysis is used on historical data to quantify errors brought on by changes in data distribution. The plan works, and the most accurate results are obtained using the K-NN algorithm at the conclusion. As additional tools for identifying covert cyberattacks, researchers developed the algorithm for extremely randomized trees and kernel principal component analysis (Acosta et al., 2020). The SVMLDT was employed to find issues with smart grids. A dynamic load rejection scheme guards against denial-of-service attacks; corrective actions are taken when necessary. The pulse, replay trip, and replay types of data integrity attacks are all considered in a new framework for identifying and preventing anomalies (Ravikumar & Govindarasu, 2020).

Consequently, attacks are classified with a 96.5% accuracy rate using machine learning algorithms like KNN and DT. A cyber-physical anomaly detection system can locate data integrity threats and communication errors. A classification model can be produced with the help of the machine learning algorithm DT and variation mode decomposition. The functionality of CPADS is tested and evaluated using a typical IEEE 39 bus system. FDI attacks using machines with a high learning rate and ensemble learning capabilities. A focal-loss-light GBM ensemble classifier is built using optimized feature sets that automatically label FDIA (Mazhar et al., 2022) behavior to identify FDIA (Cao et al., 2020). Extreme learning machines perform better when their weights are set with a Gaussian random distribution, as shown (Wu et al., 2020). The state estimation process is hampered by FDI and DoS attacks, which can be located using a hierarchical clustering technique. The process is sped up and made more accurate through Kalman filters (Aflaki et al., 2021). The threat is moved using the DT algorithm.

### Challenges of artificial intelligence in smart grids

Sizeable conventional power systems are often analyzed and controlled using numerical calculations and physical modelling. The transition from the traditional to the new power grid and the development of smart grids that mainly rely on renewable energy and micro-networks have made the environment even more unpredictable and complicated. In the meantime, using new smart grid technology is uncertain because the existing energy system is supported by traditional infrastructure. The communication network must manage enormous amounts of frequently changing data because it relies on power systems. For intelligent grids, this is still a challenge. Additionally, researchers are still working to ensure AI systems’ stability, dependability, and online functionality (Cherifi & Hamami, 2018). Even though there are numerous information approaches to the problems facing the smart grid, there are still several significant problems, such as those listed below.

### Making use of renewable energy.

Smart networks must include a sizable part of renewable energy. However, renewable energy is unpredictable and challenging to measure due to its power output changing frequently and quickly. This leads to several serious issues.

### Data security and confidentiality.

Since smart grid systems involve a variety of devices and two-way communication, they are more vulnerable to hacking than traditional power grids since they are readily available to those who wish to harm. The last part showed how different security techniques had been created to quickly identify cybersecurity threats such as fake data injection, system data theft, and electricity theft, among others (Mazhar et al., 2023b). The current smart grid is vulnerable to various dangers because of how its network protocols, operating systems, and physical devices function. Performance and security are exchanged in current AI-based smart grid security systems.

### Rapid data analysis and storage.

Another critical challenge is enhancing the efficiency of storing and retrieving large amounts of smart grid data for AI applications.

### The ability of AI algorithms.

AI algorithms typically have a “black box” problem, which means they are difficult to understand or explain. The solution to this problem currently requires AI systems.

### AI-based algorithms’ limitations.

How AI is used in innovative grid systems varies greatly depending on how far AI technology has progressed. However, knowing the smart grid’s limitations is essential before introducing new technology.

## Risk Modeling Techniques

Electric utility companies will eventually need to base their security strategy on similar technology to keep up with these developments and avoid making AI-based hacks useless (Jakaria, Rahman & Hasan, 2019). The wide adoption of AI-based security solutions has significantly benefited endpoint security. These next-generation security solutions, as compared to traditional security, combine techniques for analyzing dynamic behavior with machine learning and intelligent automation (Noureen et al., 2019). Malicious code injection is immediately identified and stopped based on how it operates. The behavior analysis system continuously improves and learns from the consistent influx of threat data due to machine learning (Acosta et al., 2020). This shows that criminals still use older attacks to hurt businesses for billions of dollars. Here, we go over some of the most advanced SG research instruments. The approaches described above are based on the dynamic integration of technological advancements in electrical engineering, energy storage, big data analysis, information and communication technologies (ICT), wireless communication, and machine learning (Karimipour et al., 2019).

Additionally, there are many ways to handle problems because all local automation is updated. As a result, these advanced technologies can be used to protect users whose work is essential from disruptions. Since they must function even if something goes wrong, diagnostic methods are crucial in SG (Chehri, Fofana & Yang, 2021).

### CORAS method for security risk analysis

A summary of the CORAS method for security risk analysis is presented in Table 11.

**Table 11:** A summary of the various methods for evaluating risk.

Method references	Method references
Threat analysis, classification, and detection methods for wireless sensor networks	Botvinkin et al. (2014)
Temporal pattern recognition algorithms can be used to identify cyberattacks.	Kalech (2019)
Three features provide security for firewall configurations supporting cyber-physical infrastructure for data acquisition and supervisory control systems.	Li et al. (2019a)
Improve the performance of OCSVM’s SCADA intrusion detection system through ensemble techniques and social media analytics.	Maglaras, Jiang & Cruz (2016)
Assessment of weaknesses	Cherifi & Hamami (2018)
Malicious programs and virtual environments in a secure SCADA environment hosted in the cloud	Tran & Besanger (2016)
Malicious software and simulation	Ficco, Choraś & Kozik (2017)
Replay and pre-distribution key scheme for simulation and malicious software (Malware).	Pramod et al. (2019)
The eighth technique is crucial for transmitting and expanding the spread of SCADA systems that can resist attacks and discard packets.	Tran & Besanger (2016)

### Cyber security risk assessment methods for SCADA systems

In-depth analysis is the primary goal of this study. This article examines how SCADA systems are utilized to analyze cybersecurity risks by looking at relevant content smart Grid and SCADA system attack analysis, classification, and location using wireless sensor networks (Ravikumar & Govindarasu, 2020). Searching for temporal trends is the second technique for finding cyberattacks (Mazhar et al., 2022). Firewall idea that uses CPI to safeguard SCADA systems in networks for smart grids (Cao et al., 2020). Combining ensemble approaches and social media indicators can increase the accuracy of the One-Class Class Support Vector Machine. For the IEC 60780—5—101 SCADA protocol, Method 5 (Radanliev et al., 2020) explains how to implement absolute security realistically. A SCADA-like technique as a service for interoperability of micro-network platforms The interoperability of microgrid platforms was investigated in this study in light of the growth of the smart grid. There are now many levels of interoperability, each created to meet a particular need. The main goal of this study was to present a feasible hybrid cloud-based private SCADA architecture that met various requirements for micro-network platform interoperability while taking security standards into account. Micro-network interoperability allows academic institutions to share and exchange data, pool resources, and eventually borrow related infrastructure for on- or off-site research (Aflaki et al., 2021). A platform for critical infrastructure vulnerability analysis simulation and cybersecurity (Ravikumar & Govindarasu, 2020).

The pre-distribution of keys for SCADA systems that recognize shared licenses (Mazhar et al., 2022). Using the internet, a mobile *ad hoc* network (MANET), and wireless sensors to create an impenetrable SCADA system (Cao et al., 2020). An analysis of a smart grid’s vulnerability to assaults that change load distribution using cascading dynamics (Wu et al., 2020). Put a lot of faith in the SCADA IoT-based industrial control system to ensure its functionality (Aflaki et al., 2021). A SCADA intrusion detection strategy based on optimization (Cherifi & Hamami, 2018).

### Mitigating the risk of cyber attack on smart grid systems

To find smart grid system weaknesses and potential threats, engineers, IT managers, users, and security managers must work together more. This will help them avoid the cyber risks of today. Companies should consider alternative strategies as they plan for future cybersecurity developments. Planning and making repeated attempts to keep ahead of known threats is essential. The creation of an efficient cyber defense system must be continuous. Companies in the electricity sector need to develop a well-thought-out plan with a strong basis and straightforward steps. Traditional layered cybersecurity techniques are insufficient because they can only identify and stop low-level threats. On the other hand, modern cyber threats are designed to make it challenging for traditional security measures to counter them.

They achieve this by giving detection systems instructions on deleting their defenses. Even the most effective security measures can be overcome by threats of secret attacks by those with authorization. Cybersecurity solutions may give you the knowledge to make more informed choices about defending your smart grid from cyberattacks using AI and reducing big data analytics. They can help the power provider detect threats more quickly by monitoring the cyber world with the speed and accuracy that only computers can. To filter out harmful communications in reaction to an attack, solutions like antivirus, EDR systems, firewalls, and data loss prevention are examples of those that already use AI. Due to the growing number of vulnerabilities, the difficulty in determining their seriousness, and the difficulty in automatically selecting and distributing patches, operational teams are concerned about vulnerability management. Only a tiny portion of the millions of vulnerabilities yearly found and reported are used by bad actors. Some systems also have boundary walls to keep them safe. As a result, developers of vulnerability management systems are adding AI to their creations more frequently. Artificial intelligence is used in vulnerability management to speed up and improve procedures, including discovering assets, searching for vulnerabilities, evaluating risk based on threat data, prioritizing solutions, and deploying them.

The CORA’s method for security risk analysis is shown in Fig. 18.

Passwords and login names are insufficient for secure remote access. In this case, a VPN should be used to establish an encrypted connection (Noureen et al., 2019). These facts show that no security solution works for all firms, including energy providers (Sundararajan et al., 2018; Cai et al., 2017; Rice & AlMajali, 2014). Instead, each company must customize security protocols to meet its unique needs. This strategy is the only way to guarantee that everyone is sufficiently protected.

The first step is to control the flow of information. You can achieve this by using a firewall or another device that controls the direction of the protocols used by IT and OT systems when they communicate. If IT should only contact OT, limiting communication to the HTTPS protocol makes sense. As a result, attacks that rely on server message blocks are prevented (Sundararajan et al., 2018). Start by conducting a thorough risk analysis that considers internal and external threats. To develop security guidelines and risk mitigation strategies, experts will identify the weak spots (Cai et al., 2017). Every utility needs to create a security strategy and protocol. This is so because a company’s cybersecurity policy specifies employees’ rules. A utility’s security policy communicates to staff members, suppliers, and other authorized users the company’s expectations for protecting electronic information and assets and the consequences of breaking the rules. Examining your foundation once or twice a year is one way to keep it in good shape. It is crucial to choose a cybersecurity solution that complies with global norms and take the necessary steps to implement this plan. The electrical industry may benefit significantly from deep packet inspection, also called a “deep look” into data communication.

Additionally, it has a considerable impact on industry productivity. As a result, new communication protocols and measurements that vary from the norm can be found quickly. This reduces the possibility of damage by enabling quick response to a slowly spreading attack or error. The typical operation of the system is known after some initial understanding. An alert is set off when something deviates from the norm. Such a discrepancy may be caused by a virus assault, a broken sensor, or a service technician using a brand-new laptop. Figure 19 shows the mitigating the risk of cyber-attacks on smart grid systems (Rice & AlMajali, 2014).

## Conclusion

The transition to smart grids represents a crucial step in modernizing our electrical infrastructure to meet the growing demands of society. These grids enable more efficient energy management, promote the use of renewable energy sources, and contribute to the reduction of carbon dioxide emissions. However, with their increasing sophistication and interconnectivity, smart grids have become potential targets for increasingly sophisticated cyberattacks.

Despite modern security technologies, cybersecurity challenges remain significant in the context of smart grids. Attackers are constantly developing new methods to compromise these critical systems. Establishing flexible approaches to assess datasets and identify hidden risks is imperative. This is where AI and big data come into play.

AI, machine learning, and deep learning have made significant strides in recent years, enabling the analysis of vast amounts of data adaptively. Machine learning can detect new attacks and unexpected behaviors thanks to adaptable baseline behavior models. By combining this new data with existing datasets and predictive analytics techniques, we can significantly enhance the security of smart grids. Using big data and AI in the context of smart grids also offers the opportunity to understand the current situation better and develop potential solutions for cybersecurity issues. This enables a proactive response to threats and continuous improvement of security.

This article highlights the different types of attacks that smart grids face and the specific challenges AI poses in this field. It also explores the use of big data in smart grids and its potential application in other areas, such as healthcare. Finally, the article proposes a solution to address the security challenges of smart grids using AI and big data methods. By integrating AI and cloud computing, it is possible to develop a fully autonomous and self-learning smart grid system, enhancing security and reliability while reducing downtime.

In conclusion, smart grids are essential to meet the growing energy needs of our society while promoting sustainability and reducing greenhouse gas emissions. However, their security remains a significant challenge. AI and big data offer promising solutions to strengthen the cybersecurity of smart grids by enabling early threat detection and proactive response. Looking ahead, the integration of AI and cloud computing, as well as the development of transfer learning techniques, pave the way for even more advanced and resilient smart grids.

## Future Work

Smart grids aim to develop an efficient, cost-effective, conscious, flexible, and responsive system. Here are a few possible future enhancements for smart grid technologies.

Since it may boost security and dependability while reducing failures, integrating artificial intelligence and cloud computing will become critical in developing a full self-learning smart grid system.

Transferring processed data to the cloud is an alternative to fog computing. As an alternative, some processing is done locally. Fog computing offers on-demand processing resources, which is the basis of its many benefits (*e.g.*, energy efficiency, scalability, flexibility). As data levels rise, fog computing will play a bigger role in the future smart grid.

The lack of label data that can be used for transfer learning is one of the significant problems with smart grid research. Transfer learning lowers the amount of training data needed, assisting researchers in addressing the issue of insufficient data.

As fog computing and the expansion of the 5G network make it possible, it is becoming increasingly crucial to predict how people will use power systems.

As fog computing and the expansion of the 5G network make it possible, it is becoming increasingly crucial to predict how people will use power systems. Understanding human behavior and electricity consumption patterns can significantly enhance consumer demand responsiveness.

## Additional Information and Declarations

### Data Availability

The following information was supplied regarding data availability: This is a literature review.
